# p-STAT3-elevated E3 ubiquitin ligase DTX4 confers the stability of HBV cccDNA by ubiquitinating APOBEC3B in liver

**DOI:** 10.7150/thno.99407

**Published:** 2024-09-16

**Authors:** Lina Zhao, Hongfeng Yuan, Yufei Wang, Chunyu Hou, Pan Lv, Huihui Zhang, Guang Yang, Xiaodong Zhang

**Affiliations:** State Key Laboratory of Druggability Evaluation and Systematic Translational Medicine, Tianjin Key Laboratory of Digestive Cancer, Department of Gastrointestinal Cancer Biology, Tianjin Cancer Institute, Liver Cancer Center, Tianjin Medical University Cancer Institute and Hospital, National Clinical Research Center for Cancer, Tianjin's Clinical Research Center for Cancer, Tianjin 300060, China.

**Keywords:** HBV cccDNA, DTX4, APOBEC3B, Cucurbitacin I, PEG-IFN α

## Abstract

**Background:** Clinically, the persistence of HBV cccDNA is the major obstacle in anti-HBV therapy. However, the underlying mechanism of HBV cccDNA is poorly understood. The transcriptional factor STAT3 is able to activate HBV replication in liver.

**Approach & Results:** RNA-Seq analysis demonstrated that cucurbitacin I targeting STAT3 was associated with virus replication in liver. HBV-infected human liver chimeric mouse model and HBV hydrodynamic injection mouse model were established. Then, we validated that cucurbitacin I effectively limited the stability of HBV cccDNA and HBV replication in cells, in which cucurbitacin I enhanced the sensitivity of pegylated interferon α (PEG-IFN α) against HBV *via* combination *in vitro* and *in vivo*. Mechanistically, we identified that cucurbitacin I increased the levels of APOBEC3B to control HBV cccDNA by inhibiting p-STAT3 in cells, resulting in the inhibition of HBV replication. Moreover, RNA-Seq data showed that E3 ubiquitin ligase DTX4 might be involved in the events. Then, we observed that HBV particles could upregulate DTX4 by increasing the levels of p-STAT3 *in vitro* and *in vivo*. The p-STAT3-elevated DTX4/male-specific lethal 2 (MSL2) independently and synergistically enhanced the stability of HBV cccDNA by facilitating the ubiquitination degradation of APOBEC3B in cells, leading to the HBV replication.

**Conclusions:** p-STAT3-elevated DTX4 confers the stability of HBV cccDNA and HBV replication by facilitating the ubiquitination degradation of APOBEC3B. Cucurbitacin Ⅰ effectively enhances the sensitivity of PEG-IFN α in anti-HBV therapy by inhibiting the p-STAT3/DTX4/MSL2/APOBEC3B signalling. Our finding provides new insights into the mechanism of HBV cccDNA. The p-STAT3 and DTX4/MSL2 might serve as the therapeutical targets of HBV cccDNA.

## Introduction

Hepatitis B virus (HBV) is a global health problem that can lead to cirrhosis and hepatocellular carcinoma (HCC) 1-4. Clinically, pegylated interferon (PEG-IFN) and nucleoside analogues could prevent the disease, but failed to eradicate the virus 5-9. The persistence of HBV covalently closed circular DNA (HBV cccDNA) in the liver is the main reason and obstacle in eradicating HBV and in the reactivation of HBV replication in immunosuppressed patients 10-14. HBV cccDNA is accumulated in nucleus as a chromatin-like cccDNA minichromosome assembled by histones and non-histones proteins 15. Accumulating evidence suggests that the host epigenetic modulations, including DNA methylation and DNA-bound histone modifications, play vital roles in the cccDNA transcriptional activity 16, 17. The histone modifications on cccDNA affect HBV transcription. The H3 and H4 histones, HBc, HBx, the host transcription factors, and the epigenetic modification enzymes such as P300, HAT1, SIRT1, PRMT1/5, IFI16, HDAC1 and HDAC3 have been associated with cccDNA transcription 16-21. Apolipoprotein B mRNA editing enzyme, catalytic polypeptide-like 3A (APOBEC3A) and apolipoprotein B mRNA editing enzyme, catalytic polypeptide-like 3B (APOBEC3B) play an important role in the regulation of HBV cccDNA stability 22-25. Mechanically, HBV core proteins mediate the interaction of APOBEC3A and APOBEC3B with HBV cccDNA, resulting in the cytidine deamination, apurinic/apyrimidinic site formation, and finally cccDNA degradation that prevents HBV reactivation 26. It has been reported that the stabilization of hypoxia inducible factor-1 (HIF1α) impairs APOBEC3B mediated by nuclear factor kappa-B (NF-κB), which is important for the elimination of HBV cccDNA 22. Our group previously reported that HBx-elevated Male-specific lethal 2 (MSL2) modulates HBV cccDNA by inducing degradation of APOBEC3B 23. Long non-coding RNA HULC activates HBV by modulating HBx/STAT3/miR-539/APOBEC3B signalling in HBV-related hepatocellular carcinoma 27. Interferon-α (IFN-α) displays potent antiviral activities by inducing IFN-stimulated genes (ISGs), and the critical role of IFN-α-induced signalling pathways has been demonstrated in the host antiviral responses 28, 29. Moreover, accumulating evidence suggests that IFN-α inhibits the levels of the HBV cccDNA 30-37, and it has been reported that IFN-α up-regulates APOBEC3A cytidine deaminases 26. However, the underlying mechanism of HBV cccDNA is poorly understood.

The JAK/STAT axis is implicated in cancer, inflammation, and immunity. The canonical pathway involves the activation of JAK following ligand binding to cytokine receptors. The activated JAKs then phosphorylate STAT proteins, leading to their dimerization and translocation into the nucleus. In the nucleus, STATs act as transcription factors with pleiotropic downstream effects 38. Cucurbitacin I is a plant-derived natural tetracyclic triterpenoid compound that shows an anticancer effect via inhibiting the JAK2-STAT3 signalling pathway 39-41. Cucurbitacin I-treated macrophages reduce the migration of cancer cells by inhibiting M2 polarization, highlighting the potential of cucurbitacin I as a therapeutic drug targeting M2-like macrophages to reduce cancer cell metastasis 42. Cucurbitacin I targeting p-STAT3 displays anticancer effects by inducing apoptosis of HepG2 cell lines and regulating JAK/STAT3, MAPK/ERK and AKT/mTOR signalling pathways 43. However, whether cucurbitacin I is involved in the modulation of HBV cccDNA remains unclear.

The human Deltex (DTX) protein family consists of five members, namely DTX1, DTX2, DTX3, DTX3L and DTX4, which have the function of E3 ubiquitin ligase 44, 45. DTX4 is involved in the pathways such as NOTCH2 activation and nuclear and cytoplasmic sensors that transmit signals to pathogen-associated DNA 46. DTX4 leads to ubiquitin-mediated proteasome degradation of TBK1, thereby inhibiting interferon signalling 47. During the reactivation of Epstein-Barr virus, the RNA m6A demethylase AlkB homolog 5 (AlkB homolog 5, ALKBH5) was significantly downregulated, resulting in enhanced methylation of the cellular transcripts DTX4 and Tyrosine Kinase 2 (TYK2) 48. Thus, TYK2 mRNAs degradation and DTX4 mRNAs translation efficiency are higher, leading to a weakening of interferon signalling, which induces the progression of viral cleavage replication 48. However, whether DTX4 is involved in the modulation of HBV cccDNA levels and HBV replication is not well documented.

In this study, we try to identify the targets and inhibitor of HBV cccDNA. Interestingly, we observed that cucurbitacin I targeting p-STAT3 could limit the stability of HBV cccDNA and HBV replication by inhibiting the ubiquitination degradation of APOBEC3B, in which p-STAT3 transcriptionally activated E3 ubiquitin ligase DTX4/MSL2 in cells. Moreover, cucurbitacin I could effectively enhance the sensitivity of PEG-IFN α in anti-HBV therapy by inhibiting the p-STAT3/DTX4/MSL2/APOBEC3B signalling. Our findings provide new insights into the mechanism by which DTX4 modulates the stability of HBV cccDNA.

## Methods

### Patient samples

The clinical and virological characteristics of the patients enrolled in this study are summarized in Supplementary Table 1 and 2. Liver tissue samples were obtained by percutaneous needle biopsy, immediately frozen in liquid nitrogen, and stored at -80 °C. Patients provided written informed consent for the use of their tissue for research purposes after surgery, and the Institute Research Ethics Committee at the Tianjin Medical University Cancer Institute and Hospital approved the study protocol (approval number: EK20240175). The HCC peritumoral tissue samples utilized in this study were obtained from Tianjin Medical University Cancer Institute and Hospital (Tianjin, China) after surgical resection. All patients provided written consent for the use of their tissues for research purposes after surgery. All research was conducted in accordance with both the Declarations of Helsinki and Istanbul. All study procedures were in compliance with the regulations of the Institute of Research Ethics Committee at Tianjin Medical University Cancer Institute and Hospital (Tianjin, China).

### Animals

Hydrodynamic injection (HDI) of HBV was performed with the pAAV HBV 1.2 plasmid. Male mice were divided into 4 groups with 6 mice in each group: DMSO, Cucurbitacin I (0.5 mg/kg, intraperitoneal injection), PEG-IFN α (25 μg/kg, subcutaneous injection) and Cucurbitacin I (0.5 mg/kg, intraperitoneal injection) + PEG-IFN α (25 μg/kg, subcutaneous injection). Serum samples were collected from mice in the HBV infection groups of mice 0 week (Day 0, 1 week after virus inoculation), 1 week (Day 7), 2 weeks (Day 14), and 3 weeks (Day 21) and 4 weeks (Day 28) after administration. Then, the animals were sacrificed, and the liver and blood were collected for RT-qPCR analysis, immunostaining or ELISA analysis 4 weeks after administration.

Human liver chimeric mice were generated by VITALSTAR Inc. (Beijing, China) 49. The mice were then sacrificed 8 weeks after virus inoculation. The serum HBV DNA titre, HBsAg and HBeAg in the mice was determined before sacrifice. Information on the human liver chimeric mice is shown in Supplementary Table 3. All animal experiments were in compliance with the Guide for the Care and Use of Laboratory Animals (NIH publications 86-23 revised 1985) and were performed according to the institutional ethical guidelines. The Institute Research Ethics Committee at Tianjin Medical University Cancer Institute and Hospital approved the study protocol (approval number: NSFC-AE-2024229).

### Plasmids and reagents

All plasmids used in this study were constructed by Tsingke Biotech Co., Ltd. unless specifically stated otherwise. To construct the DTX4-WT/MSL2-WT reporter plasmid, the 5' flanking region of transcription start site (-2,000 to +200 bp) of *DTX4*/*MSL2* gene was inserted into the pGL3-basic vector. To test binding specificity, the sequence in the 5' flanking region (-949 to -939 bp/-852 to -842 bp) of *DTX4*/*MSL2* gene that interacted with transcription factor STAT3 was mutated from TTTTCCAGAAA to AAAAGGTCTTT/CTTTTGAGAAG to GAAAACTCTTC for construction of the mutant vector (pGL-DTX4/MSL2 promoter mut). The small interfering RNAs targeting the indicated genes (Supplementary Table 4) and the control small interfering RNAs were purchased from Sangon Biotech (Shanghai, China). The plasmids used for transfection are listed in Supplementary Table 5. The antibodies used are listed in Supplementary Table 6. Cucurbitacin I was purchased from MedChemExpress LLC (Shanghai, China). The recombinant murine interferon α is expressed in yeast system. PEG-IFN α-2b solution (0.36 mg/mL), which is derived from human, which were provided by Xiamen Amoytop Biological Engineering Co., Ltd. (Xiamen, China).

### Southern blot analysis

HBV infection was performed as previously described 20, 50, 51. The extracted DNA sample from HepG2-NTCP cells was separated through 1.0% agarose gel using a Southern blot kit (Roche, USA), blotted onto a nylon membrane, and hybridized with DIG-labelled probes of linear HBx DNA fragments.

### Statistical analysis

The data are presented as the mean ± standard deviation (SD) of at least three replicate experiments. Statistical analyses were performed using Prism software (version 8.01, GraphPad). To compare the means of two groups with normal (or approximately normal) distributions, an unpaired *t* test was applied. When multiple *t* tests were used to compare data between two groups, adjusted *P* values were computed with the Holm-Sidak method. To compare means among >2 groups, we used one-way analysis of variance (ANOVA) with correction for multiple comparisons (Dunnett's test, Tukey's test, or the Sidak correction). Statistical significance was assumed for *P<0.05, **P<0.01, and ***P<0.001.

## Results

### Cucurbitacin I attenuates the HBV replication and enhances the sensitivity of PEG-IFN α against HBV

**Figure 1:**
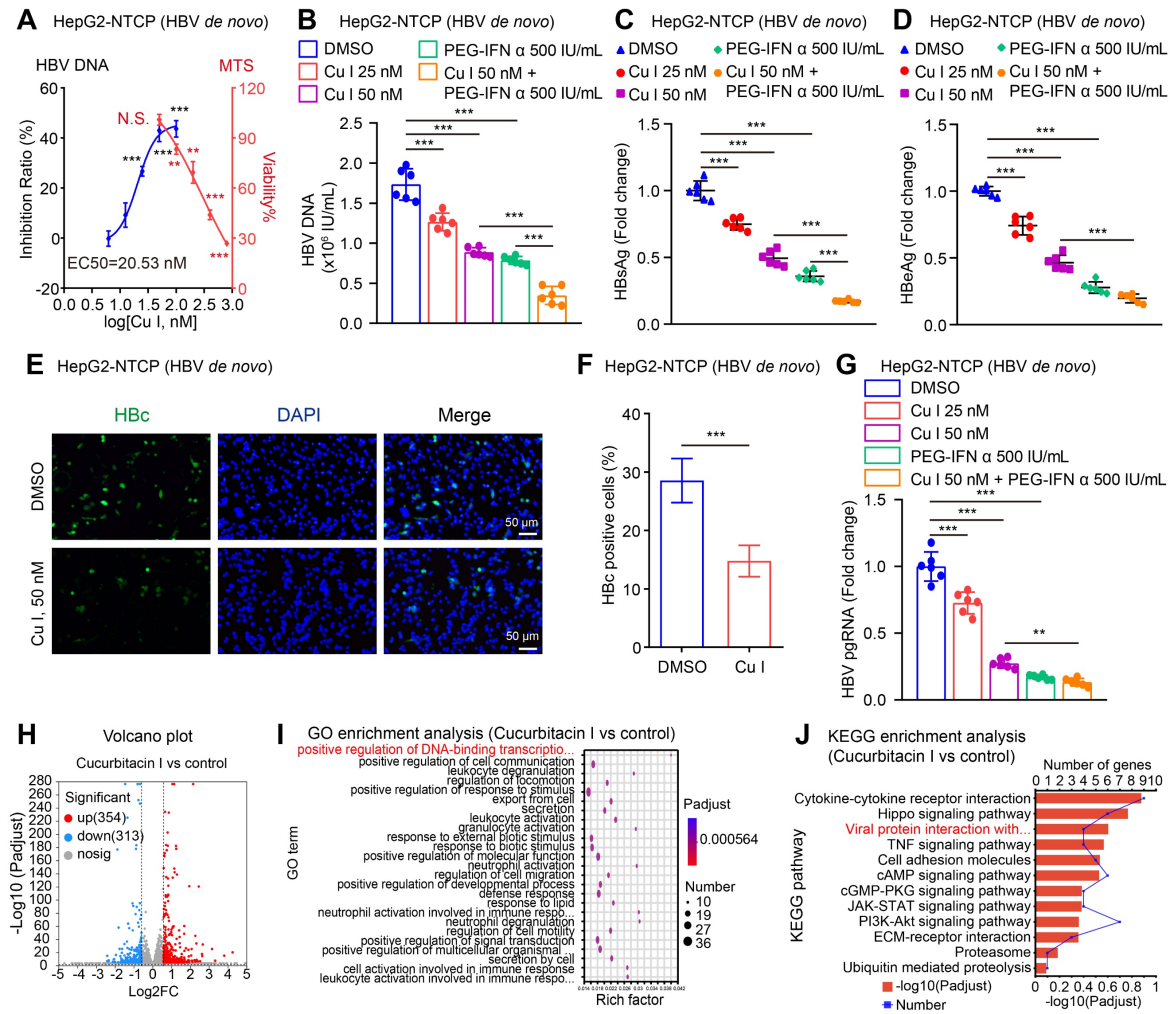
Cucurbitacin I attenuates the HBV replication and enhances the sensitivity of PEG-IFN α against HBV *in vitro*. (A-G) HepG2-NTCP cells were subjected to HBV infection (MOI = 500) and treated with factors as indicated at 1 and 4 dpi (days post-infection). Analysis was performed 7 days post-infection. The levels of HBV DNA were measured by real-time PCR in the supernatant of HBV-infected HepG2-NTCP cells treated with cucurbitacin I (A). The viability of HBV-infected HepG2-NTCP cell treated with cucurbitacin I was evaluated by the MTS assays (A). The HBV DNA content was tested by real-time PCR (B). The levels of HBsAg and HBeAg were validated by ELISA as appropriate in the supernatants (C, D). The HBc expression levels were examined by immunofluorescence (E). ImageJ was used to quantitate the protein expression levels in the immunofluorescence analysis (F). The levels of HBV pgRNA were determined by RT-qPCR in the cells (G). (H) The Volcano plot of the differentially expressed genes (DEGs) that cucurbitacin I has a significant effect on HBV-infected hepatoma cells analyzed by RNA-Seq (the screening criteria were |FC| ≥1.5 & Padj<0.05). (I, J) The GO/KEGG enrichment analysis of DEGs between the control and cucurbitacin I groups (*n*=3). The mean ± SD of at least three experiments is shown. Statistically significant differences are indicated as follows: ^*^P < 0.05, ^**^P < 0.01, ^***^P < 0.001. Abbreviation: N.S., not significant, Cu I, Cucurbitacin I.

It has been reported that HBV activates STAT3 signalling in liver to support virus replication 52, 53. Accordingly, we supposed that cucurbitacin I targeting p-STAT3 might play a role in anti-HBV therapy. Then, we examined the effect of cucurbitacin I on HBV in cells. Notably, we observed that cucurbitacin I restricted the HBV DNA replication, in which EC50 of cucurbitacin I for HBV DNA inhibition was 20.53 nM (Figure 1A). Meanwhile, cucurbitacin I had no toxic effect on HepG2-NTCP cells when it was less than or equal to 50 nM (Figure 1A). The levels of HBV replication-related indexes, including HBV DNA, HBsAg, HBeAg, HBc and HBV pgRNA (Figure 1B-G) were remarkably decreased by cucurbitacin I in a dose-dependent manner in HBV-infected HepG2-NTCP cells. Moreover, cucurbitacin I enhanced the sensitivity of PEG-IFN α against HBV (Figure 1B-C). To better understand the effect of cucurbitacin I on HBV, we performed RNA-Seq analysis in HBV-infected HepG2-NTCP cells treated with cucurbitacin I. The Volcano plot analysis showed that cucurbitacin I could regulate 667 genes, including 354 up-regulated genes and 313 down-regulated genes in a variety of biological functions (Figure 1H and S1A). GO enrichment analysis indicated that cucurbitacin I was related to the positive regulation of DNA-binding transcription factor activity, positive regulation of cell communication, and leukocyte degranulation (Figure 1I).

**Figure 2:**
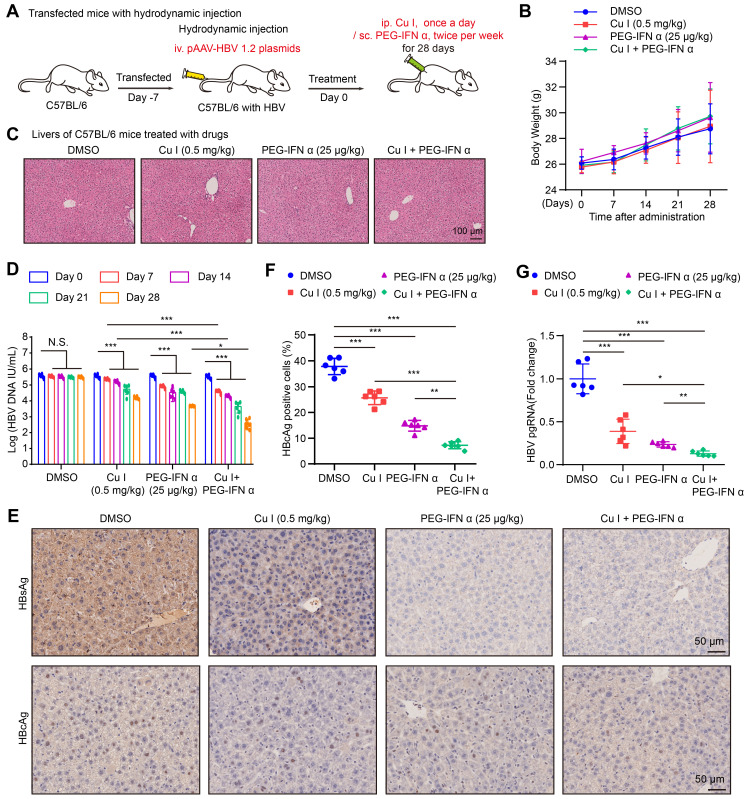
Cucurbitacin I attenuates the HBV replication and enhances the sensitivity of PEG-IFN α against HBV *in vivo*. (A) Schematic representation of mouse experimental process. (B) The mice were administered by cucurbitacin I (0.5 mg/kg) once a day and PEG-IFN α (25 μg/kg) twice per week for 4 weeks. Serum samples were collected before treatment (day 0) and days 7, 14, 21, and 28 after treatment. A comparative evaluation of changes in the body weight of HDI mice treated with the indicated compounds. (C) Pathological characteristics were observed by H&E staining in the liver tissues from mice treated with the indicated compounds. Scale bars: 100 μm. (D) The levels of HBV DNA in the serum of HDI mice were tested by qPCR. (E) Immunohistochemical staining for HBsAg and HBcAg were conducted in liver tissues. Scale bars: 50 μm. (F) The HBcAg expression levels in liver tissue samples were quantified by using the software of ImageJ. (G) The levels of HBV pgRNA in liver tissues from HBV-infected mice were measured by RT-qPCR. The mean ± SD of at least three experiments is shown. Statistically significant differences are indicated as follows: ^*^P < 0.05, ^**^P < 0.01, ^***^P < 0.001. Abbreviation: N.S., not significant, Cu I, Cucurbitacin I.

KEGG pathway enrichment analysis and annotation analysis revealed that cucurbitacin I was associated with several biological aspects, such as human diseases, metabolism, environmental information processing, cellular processes, and organismal systems, specifically including cytokine-cytokine receptor interaction, Hippo signalling pathway, viral protein interaction with cytokine and cytokine receptor (Figure 1J and S1B), suggesting that cucurbitacin I might be associated with the viral infection. To verify the effect of cucurbitacin I on HBV replication *in vivo*, we constructed a HDI mouse model of HBV infection (Figure 2A). The weights of the experimental mice and pathological characteristics of the liver tissues showed that 0.5 mg/kg cucurbitacin I and 25 μg/kg PEG-IFN α had no toxic effect on mice (Figure 2B-C). As expected, the levels of HBV DNA in the serum from the mice treated with cucurbitacin I were significantly decreased from day 7 relative to day 0 (Figure 2D). IHC staining revealed that the levels of HBsAg and HBcAg were markedly reduced in the liver tissues from mice treated with cucurbitacin I compared to those from control group (Figure 2E-F and S2A). Fluorescent staining was used to validate the levels of HBcAg in the system (Figure S2B-C). RT-qPCR indicated that HBV pgRNA were obviously decreased in the liver tissues treated with cucurbitacin I comparison with those from the control group (Figure 2G). In addition, compared with PEG-IFN α alone, the levels of HBV DNA, HBsAg, HBcAg and HBV pgRNA were significantly decreased in the group of cucurbitacin I combined with PEG-IFN α (Figure 2D-G and S2A-C), suggesting that cucurbitacin I restricts HBV replication and enhances the sensitivity of PEG-IFN α against HBV *in vivo*. Thus, we conclude that cucurbitacin I targeting p-STAT3 attenuates the HBV replication and enhances the sensitivity of PEG-IFN α against HBV in liver.

### Cucurbitacin I enhances the sensitivity of PEG-IFN α against HBV cccDNA by elevating the levels of APOBEC3B

**Figure 3:**
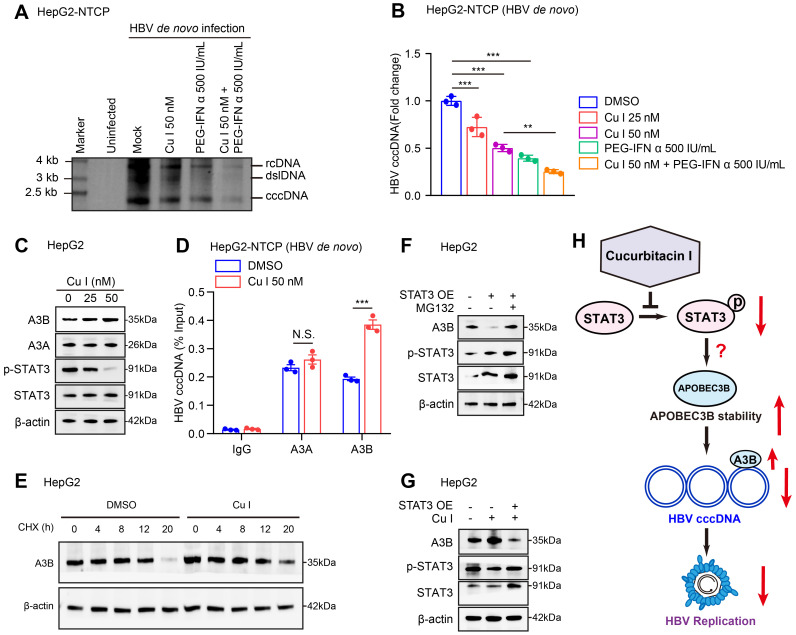
Cucurbitacin I enhances the sensitivity of PEG-IFN α against HBV cccDNA by elevating the levels of APOBEC3B. (A, B) HepG2-NTCP cells were subjected to HBV infection (MOI = 500) and treated with factors as indicated at 1 and 4 dpi (days post-infection). Analysis was performed 7 days post-infection. The HBV cccDNA content was tested by Southern blot analysis (A) and qPCR (B). (C) The effect of cucurbitacin I on the expression levels of APOBEC3A and APOBEC3B was detected by Western blot analysis in HepG2 cells treated with IL-6 to induce STAT3 activation for 16 hours. (D) The effect of cucurbitacin I on the binding of APOBEC3B to HBV cccDNA was evaluated by ChIP-qPCR in HBV-infected HepG2-NTCP cells. (E) HepG2 cells were treated with DMSO or cucurbitacin I 48 h followed by cycloheximide (CHX, 100 μg/mL) treatment for the indicated time. Cells treated with IL-6 to induce STAT3 activation for 16 hours. The protein levels of APOBEC3B were examined by Western blot analysis. (F) HepG2 cells were transfected with pcDNA3.1 STAT3 (2 μg) for 48 h and then treated with MG132 (2 mM) for 24 h. Cells treated with IL-6 to induce STAT3 activation for 16 hours. The protein levels of APOBEC3B and STAT3 were examined by Western blot analysis in the cells. (G) Western blot analysis was performed to determine whether the effect of cucurbitacin I on APOBEC3B protein expression through STAT3 in HepG2 cells treated with IL-6 to induce STAT3 activation for 16 hours. (H) Schematic diagram of the mechanism of cucurbitacin I reducing the stability of HBV cccDNA by inhibiting the proteasome degradation of APOBEC3B. The mean ± SD of at least three experiments is shown. Statistically significant differences are indicated as follows: ^**^P < 0.01, ^***^P < 0.001. Abbreviation: N.S., not significant, CHX, Cycloheximide, Cu I, Cucurbitacin I, A3A, APOBEC3A, A3B, APOBEC3B.

Next, we tested the effect of cucurbitacin I on HBV cccDNA in cells. Southern blot analysis and qPCR assays verified that cucurbitacin I could decrease the levels of HBV cccDNA and enhance the sensitivity of PEG-IFN α against HBV cccDNA in HBV-infected HepG2-NTCP cells (Figure 3A-B). Given that the APOBEC3A and APOBEC3B contributed to the HBV cccDNA degradation 26, we further identified the effect of cucurbitacin I on APOBEC3A and APOBEC3B in cells. Our data showed that cucurbitacin I had no significant effect on the mRNA expression of APOBEC3A and APOBEC3B (Figure S3A), but increased the protein expression of APOBEC3B in a dose-dependent manner (Figure 3C and S3B), but not APOBEC3A in cells, in which we validated that cucurbitacin I could effectively decrease the levels of p-STAT3, implying that a factor modulated by cucurbitacin I affects APOBEC3B through degradation. Furthermore, ChIP-qPCR assay demonstrated that cucurbitacin I enhanced the interaction of APOBEC3B with HBV cccDNA, but not APOBEC3A in cells (Figure 3D), suggesting that cucurbitacin I may contribute to the modulation of HBV cccDNA through APOBEC3B. The cycloheximide (CHX) chase experiments validated that p-STAT3-targeting inhibitor cucurbitacin I increased the protein stability of APOBEC3B (Figure 3E and S3C). Following the treatment with a proteasome inhibitor MG132, the STAT3-induced APOBEC3B downregulation could be reversed in the cells (Figure 3F and S3D), suggesting that APOBEC3B can be degraded in the proteasome pathway. Moreover, Western blot analysis showed that cucurbitacin I decreased the levels of p-STAT3 and increased the levels of APOBEC3B in HepG2 cells. Further, overexpression of STAT3 could rescue the levels of p-STAT3 and blocked the effect of cucurbitacin I on APOBEC3B in the system, supporting that cucurbitacin I enhances stability of APOBEC3B through p-STAT3, but the underlying mechanism is unclear (Figure 3G and S3E). Collectively, we concluded that cucurbitacin I enhances the sensitivity of PEG-IFN α against HBV cccDNA by elevating the stability of APOBEC3B in liver (Figure 3H).

### E3 ubiquitin ligase DTX4 contributes to HBV cccDNA and HBV replication

**Figure 4:**
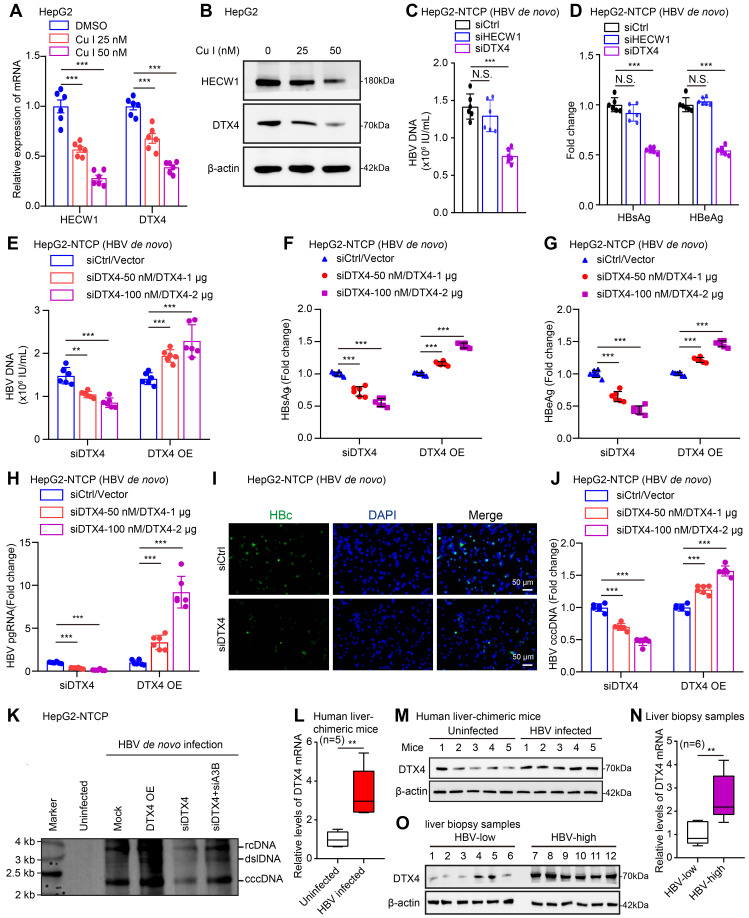
E3 ubiquitin ligase DTX4 contributes to HBV cccDNA and HBV replication. (A, B) HepG2 cells were treated with cucurbitacin I for 72 hours. Cells treated with IL-6 to induce STAT3 activation for 16 hours. The effects of cucurbitacin I on the mRNA and protein levels of HECW1 and DTX4 were detected by RT-qPCR and Western blot analysis in cells, respectively. (C-K) HepG2-NTCP cells were subjected to HBV infection (MOI = 500) and treated with factors as indicated at 1 and 4 dpi (days post-infection). Analysis was performed 7 days post-infection. The effects of HECW1 and DTX4 on HBV DNA levels were tested by real-time PCR in the supernatants (C). The levels of HBsAg and HBeAg were measured by ELISA as appropriate in the supernatants (D). The HBV DNA content was validated by real-time PCR (E). The levels of HBsAg and HBeAg were verified by ELISA as appropriate in the supernatants (F, G). The levels of HBV pgRNA were determined by RT-qPCR in the cells (H). The HBc expression levels were detected by immunofluorescence staining (I). The HBV cccDNA content was tested by qPCR (J) and Southern blot analysis (K). (L-O) The mRNA and protein levels of DTX4 were analysed by RT-qPCR and Western blot analysis in the liver tissues of HBV-infected (*n*=5) and uninfected (*n*=5) human liver chimeric mice and in liver biopsy specimens from clinical patients with a high HBV load (*n*=6) and with a low HBV load (*n*=6). The mean ± SD of at least three experiments is shown. Statistically significant differences are indicated as follows: ^*^P < 0.05, ^**^P < 0.01, ^***^P < 0.001. Abbreviation: N.S., not significant, Cu I, Cucurbitacin I, A3B, APOBEC3B.

Considering that cucurbitacin I reduces the proteasome degradation of APOBEC3B, we speculated that E3 ubiquitin ligase might be involved in the event that cucurbitacin I inhibited HBV cccDNA. By screening E3 ubiquitin ligase from the RNA-Seq analysis, we identified that cucurbitacin I significantly down-regulated the expression of E3 ubiquitin ligase HECW1 and DTX4 in HepG2 cells (Figure 4A-B and S4A). Furthermore, we screened the corresponding interference fragments with high interference efficiency in HepG2 cells (Figure S4B). The effects of DTX4 and HECW1 on HBV were tested in HepG2-NTCP cells. We observed that the interference with DTX4 significantly attenuated the levels HBV DNA, HBsAg and HBeAg levels, but interference with HECW1 failed to work in the system (Figure 4C-D and S4C), suggesting that DTX4 is closely associated with cucurbitacin I-mediated suppression of HBV in liver. We further validated the effects of DTX4 on HBV cccDNA and HBV replication in HepG2-NTCP cells. Notably, we confirmed that knockdown (or overexpression) of DTX4 decreased (or increased) the levels of HBV DNA, HBsAg, HBeAg in the cell supernatant, the levels of HBV pgRNA, HBc (Figure 4E-I and S4D) and HBV cccDNA in cells in a dose-dependent manner (Figure 4J, K and S4E), in which knockdown APOBEC3B (siA3B) could rescue the decreased levels of HBV cccDNA-mediated by siDTX4 (Figure 4K), suggesting that DTX4 contributes to the HBV cccDNA and HBV replication through APOBEC3B.

To better understand the relationship between HBV and DTX4 *in vivo*, we examined that in a model of HBV-infected human liver chimeric mice (HBV-Huhep-URG) (Figure S4F) and observed that the levels of DTX4 mRNA and protein were significantly higher in the liver of HBV-Huhep-URG mice and biopsy samples of hepatitis B patients with a high HBV load than those in the samples of control groups (Figure 4L-O), supporting that DTX4 is positively associated with HBV *in vivo*. Thus, we concluded that E3 ubiquitin ligase DTX4 contributes to HBV cccDNA and HBV replication (Figure S4G).

### DTX4/MSL2 independently and synergistically degrades APOBEC3B by ubiquitination to increase the stability of HBV cccDNA

**Figure 5:**
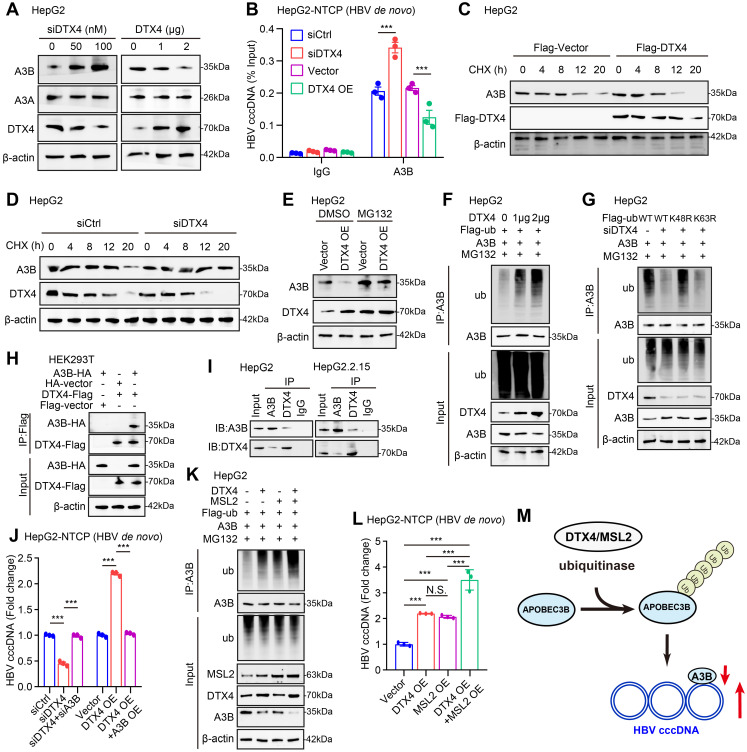
DTX4/MSL2 independently and synergistically degrades APOBEC3B by ubiquitination to increase the stability of HBV cccDNA. (A) The effects of DTX4 on the expression levels of APOBEC3A and APOBEC3B were detected by Western blot in HepG2 cells. (B) The effects of DTX4 on the binding of APOBEC3B to HBV cccDNA were examined by ChIP-qPCR in HBV-infected HepG2-NTCP cells. (C, D) HepG2 cells were transfected with Flag-vector/Flag-DTX4 (2 μg) or siCtrl/siDTX4 (100 nM) 48 h followed by CHX (100 μg/mL) treatment for the indicated time. The protein levels of APOBEC3B and Flag-DTX4 were tested by Western blot analysis. (E) HepG2 cells were transfected with Flag-DTX4 ((2 μg) for 48 h and then treated with MG132 (2 mM) for 24 h. The protein levels of APOBEC3B and DTX4 were determined by Western blot analysis in the cells. (F) Ubiquitination analysis of APOBEC3B in HepG2 cells when APOBEC3B and Flag-ubiquitin were co-transfected with DTX4 or not; cells were treated with MG132 (2 mM) for 24 h. (G) Ubiquitination analysis of APOBEC3B in HepG2 cells when APOBEC3B and Flag-ub, or Flag-ub K63R (mutation of lysine at 63 residues to arginine), or Flag-ub K48R (mutation of lysine at 48 residues to arginine) were co-transfected with siDTX4 or not. (H) The binding affinity between exogenous DTX4 and APOBEC3B was measured by a Co-IP assay in HEK293T cells transfected with DTX4 and APOBEC3B. (I) The binding affinity between endogenous DTX4 and APOBEC3B was evaluated by a Co-IP assay in HepG2 and HepG2.2.15 cells. (J) The qPCR analysis was performed to determine whether the effect of DTX4 on the levels of HBV cccDNA through APOBEC3B in HBV-infected HepG2-NTCP cells. (K) Ubiquitination analysis of APOBEC3B when APOBEC3B and Flag-ubiquitin were co-transfected with DTX4/MSL2 in HepG2 cells; cells were treated with MG132 (2 mM) for 24 h. (L) The qPCR analysis was performed to determine the effect of DTX4/MSL2 on the levels of HBV cccDNA in HBV-infected HepG2-NTCP cells. (M) Schematic diagram of the mechanism by which DTX4/MSL2 degrades APOBEC3B through ubiquitination and increases the stability of HBV cccDNA. The mean ± SD of at least three experiments is shown. Statistically significant differences are indicated as follows: ^***^P < 0.001. Abbreviation: N.S., not significant, CHX, Cycloheximide, A3A, APOBEC3A, A3B, APOBEC3B.

Given that the specific degradation mechanism of HBV cccDNA degradation through APOBEC3A and APOBEC3B 26, we try to identify the effects of DTX4 on APOBEC3A and APOBEC3B in cells. We failed to observe that DTX4 knockdown and overexpression could affect the mRNA levels of APOBEC3A and APOBEC3B in HepG2 cells (Figure S5A, B). But, we found that the knockdown (or overexpression) of DTX4 could increase (or decrease) the protein levels of APOBEC3B in a dose-dependent manner, but not APOBEC3A in the cells (Figure 5A and S5C). Further, ChIP-qPCR assays validated that knockdown (or overexpression) of DTX4 facilitated (or reduced) the levels of APOBEC3B on HBV cccDNA in HepG2-NTCP cells infected with HBV (Figure 5B and S5D), suggesting that DTX4 limits the binding of APOBEC3B to HBV cccDNA by reducing the protein levels of APOBEC3B in cells.

Next, we further identify the underlying mechanism by which E3 ubiquitin ligase DTX4 modulates APOBEC3B. CHX tracking experiments showed that the overexpression of DTX4 reduced the protein stability of APOBEC3B, while DTX4 knocking down increased that in HepG2 cells (Figure 5C-D). Moreover, we observed that the treatment with MG132, an inhibitor of protease, could rescue the decrease of APOBEC3B-mediated DTX4 overexpression in HepG2 cells (Figure 5E), suggesting that DTX4 reduces the protein stability of APOBEC3B through the proteasome pathway. The ubiquitination analysis demonstrated that E3 ubiquitin ligase DTX4 induced the ubiquitination modification of APOBEC3B in a dose-dependent manner in HepG2 cells (Figure 5F). Moreover, ubiquitination assays indicated that DTX4 predominantly preferred to assemble the K48 polyubiquitin chain on APOBEC3B protein, but not the K63-linked polyubiquitin chain (Figure 5G). Co-IP assays verified that exogenous and endogenous DTX4 interacted with APOBEC3B protein in cells (Figure 5H-I). Functionally, we revealed that APOBEC3B knockdown rescued the inhibitory effect of DTX4 knockdown on HBV cccDNA, and overexpression of APOBEC3B blocked the stimulating effect of DTX4 on HBV cccDNA in HepG2-NTCP cells infected with HBV (Figure 5J and S5E). Based on our report that HBx-elevated MSL2 modulated HBV cccDNA by inducing degradation of APOBEC3B 23, we were interested in the relationships between DTX4 and MSL2 in modulation of HBV cccDNA. The ubiquitination analysis and qPCR assays demonstrated that DTX4 and MSL2 synergistically induced the ubiquitination modification of APOBEC3B and increased the levels of HBV cccDNA in cells (Figure 5K-L). Moreover, DTX4 and MSL2 failed to affect each other without interaction in cells (Figure S5F-J). Based on the above research results, we conclude that DTX4/MSL2 independently and synergistically degrades APOBEC3B by ubiquitination to increase the stability of HBV cccDNA in a model (Figure 5M).

### STAT3 transcriptionally activates E3 ubiquitin ligase DTX4 and MSL2

**Figure 6:**
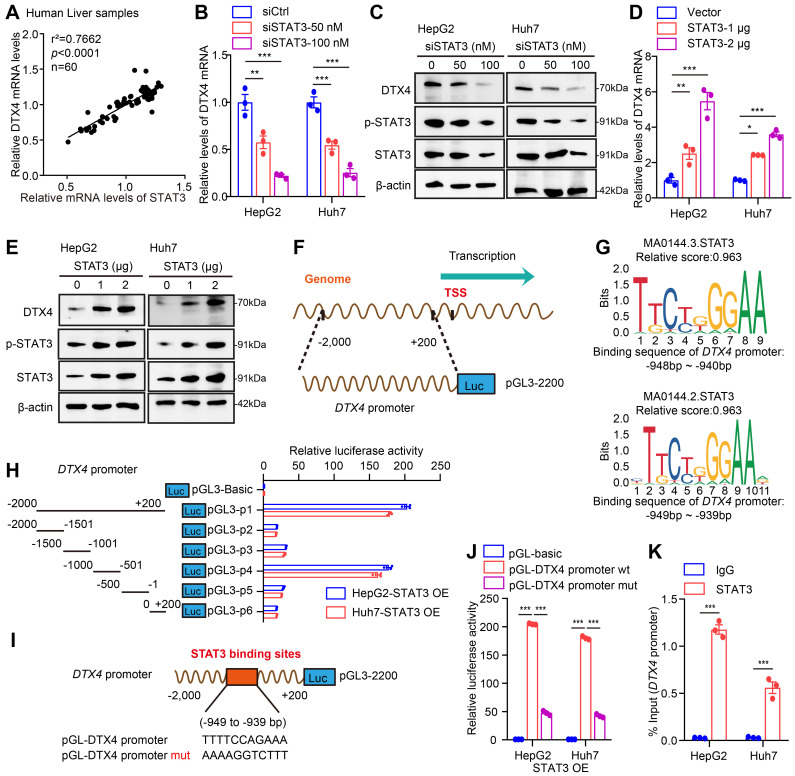
STAT3 transcriptionally activates E3 ubiquitin ligase DTX4. (A) Correlation analysis of the mRNA expression levels of STAT3 and DTX4 in paracancerous tissue of human clinical hepatocellular carcinoma samples (*n*=60). (B, C) The effects of interfering STAT3 on DTX4 mRNA and protein expression levels were detected by RT-qPCR and Western blot analysis in HepG2 and Huh7 cells treated with IL-6 to induce STAT3 activation for 16 hours. (D, E) The effects of overexpression of STAT3 on DTX4 mRNA and protein expression were verified by RT-qPCR and Western blot analysis in HepG2 and Huh7 cells treated with IL-6 to induce STAT3 activation for 16 hours. (F) Schematic illustration showing the pGL3-DTX4 promoter plasmid containing the -2,000 to +200 bp sequence of the 5' flanking region of DTX4. (G) STAT3 sequence motif logos and the binding elements in the promoter regions of *DTX4* gene were identified by JASPAR database with a relative score higher than 90%. (H) Luciferase activities of *DTX4* promoter reporter gene vectors in HepG2 and Huh7 cells transfected with STAT3 were examined by dual-luciferase reporter assays. Cells treated with IL-6 to induce STAT3 activation for 16 hours. (I) A model demonstrates the predicted STAT3 binding site at -949 to -939 bp of the *DTX4* promoter region. The generated mutant sites at the STAT3 binding site region were indicated. The wild-type *DTX4* promoter (or mutant) was inserted into the upstream of the luciferase reporter gene in the pGL3-control vector. (J) Luciferase activities of *DTX4* promoter or *DTX4* mutant promoter reporter gene vectors in HepG2 and Huh7 cells transfected with STAT3 were examined by dual-luciferase reporter assays. Cells treated with IL-6 to induce STAT3 activation for 16 hours. (K) The enrichment of STAT3 on the DTX4 region of -1,050 bp to -850 bp was examined by ChIP- qPCR assays in HepG2 and Huh7 cells treated with IL-6 to induce STAT3 activation for 16 hours. The mean ± SD of at least three experiments is shown. Statistically significant differences are indicated as follows: ^*^P < 0.05, ^**^P < 0.01, ^***^P < 0.001.

Considering that the cucurbitacin I targeting p-STAT3 significantly down-regulated DTX4, we supposed that the transcription factor STAT3 might activate DTX4 in cells. Clinical correlation analysis showed that the mRNA levels of STAT3 were positively correlated with DTX4 in paracancerous tissue of human clinical hepatocellular carcinoma samples (Figure 6A). Functionally, STAT3 knockdown downregulated the expression of DTX4 in a dose-dependent manner, while STAT3 overexpression upregulated the expression of DTX4 in a dose-dependent manner in HepG2 and Huh7 cells (Figure 6B-E and S6-1A-D). To further explore the regulatory mechanism by which STAT3 modulated DTX4, we constructed a plasmid expressing the promoter region (-2000 bp to +200 bp in the *DTX4* 5' flanking region) as a model (Figure 6F). JASPAR database analysis showed that the relative score of binding between the STAT3 recognition motif and the *DTX4* gene promoter region (from -949 to -939 bp) was as high as 0.963 (Figure 6G). Moreover, dual-luciferase reporter assays demonstrated that STAT3 significantly increased the transcriptional activity of -1000~-501 bp of the *DTX4* 5' flanking region in HepG2 and Huh7 cells (Figure 6H), but the transcriptional activity of mutant luciferase reporter mutated from -949 to -939 bp was significantly lower than that of WT group (Figure 6I-J). ChIP-qPCR analysis confirmed that STAT3 could bind to the *DTX4* promoter region in HepG2 and Huh7 cells (Figure 6K), suggesting that STAT3 as a transcription factor activates the expression of DTX4 by binding to the *DTX4* promoter region in cells.

Based on the report that HBx-elevated MSL2 modulates HBV cccDNA by inducing degradation of APOBEC3B 23, we were interested in the effect of STAT3 on MSL2 in modulation of HBV cccDNA. Correlation analysis showed that the mRNA levels of STAT3 were positively associated with those of MSL2 in liver cancer tissues of the TCGA-LIHC (liver hepatocellular carcinoma) dataset and in paracancerous tissue of human clinical hepatocellular carcinoma samples (Figure S6-1E-F). Similarly, we observed that STAT3 transcriptionally activated MSL2 as DTX4 in cells (Figure S6-1G-L). STAT3 could bind to the *MSL2* promoter region of -852 to -842 bp in HepG2 and Huh7 cells as well (Figure S6-2A-D), suggesting that STAT3 as a transcription factor stimulates the expression of MSL2 by binding to the *MSL2* promoter region. Considering our and other previous reports that HBV could upregulate STAT3 signalling in liver 53, 54, we asked whether STAT3 modulated HBV in a positive feedback manner in our system. Strikingly, we observed that HBV infection facilitated the levels of phosphorylation of STAT3 in clinical patients with a high HBV load, but not STAT3. Moreover, HBV infection was positively correlated with the levels of MSL2 and STAT3 phosphorylation, and negatively correlated with those of APOBEC3B in the tissues (Figure S6-2E-H), suggesting that HBV-elevated p-STAT3 promotes the HBV cccDNA and HBV replication by stimulating DTX4/MSL2/APOBEC3B signalling in a positive feedback manner (Figure S6-2I). Taken together, we conclude that STAT3 transcriptionally activates E3 ubiquitin ligase DTX4 and MSL2 to promote the stability of HBV cccDNA and HBV replication.

### STAT3 enhances the stability of HBV cccDNA and HBV replication through DTX4/MSL2-mediated ubiquitination of APOBEC3B

**Figure 7:**
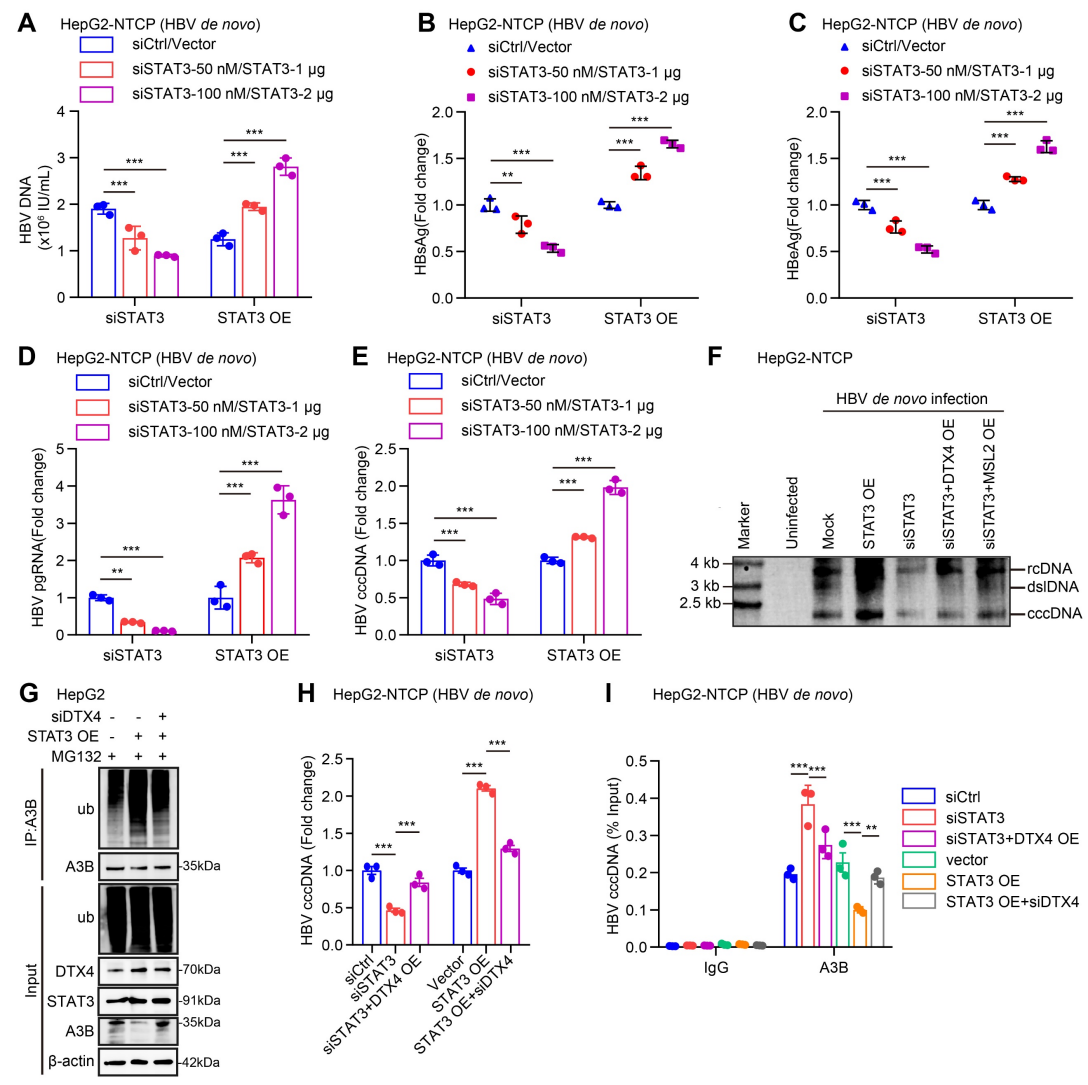
STAT3 enhances the stability of HBV cccDNA and HBV replication through DTX4/MSL2-mediated ubiquitination of APOBEC3B. (A-F) HepG2-NTCP cells were subjected to HBV infection (MOI = 500) and treated with factors as indicated at 1 and 4 dpi (days post-infection). Analysis was performed 7 days post-infection. The HBV DNA content was tested by real-time PCR (A). The levels of HBsAg and HBeAg were measured by ELISA as appropriate in the supernatants (B, C). The levels of HBV pgRNA were determined by RT-qPCR in the cells (D). The HBV cccDNA content was evaluated by qPCR (E) and Southern blot analysis (F). (G) Ubiquitination analysis of APOBEC3B in HepG2 cells when STAT3 were co-transfected with siDTX4 or not; cells were treated with MG132 (2 mM) for 24 h. Cells treated with IL-6 to induce STAT3 activation for 16 hours. (H) PCR analysis was performed to determine whether the effect of STAT3 on the levels of HBV cccDNA through DTX4 in HBV-infected HepG2-NTCP cells. (I) ChIP-qPCR analysis was performed to determine whether the effect of STAT3 on binding of APOBEC3B to HBV cccDNA micro-chromosome through DTX4 in HBV-infected HepG2-NTCP cells. The mean ± SD of at least three experiments is shown. Statistically significant differences are indicated as follows: ^**^P < 0.01, ^***^P < 0.001. Abbreviation: A3B, APOBEC3B.

Next, we focus on the investigation of effects of STAT3 on stability of HBV cccDNA and HBV replication. Our data verified that STAT3 knockdown (or overexpression) significantly decreased (or increased) the levels of HBV DNA, HBsAg, HBeAg in cell supernatant, and the levels of HBV pgRNA and HBV cccDNA in HepG2-NTCP cells-infected HBV in dose-dependent manner (Figure 7A-E, S7A-B), suggesting that STAT3 contributes to the HBV cccDNA and HBV replication. Moreover, Southern blot analysis demonstrated that STAT3 overexpression increased the stability of HBV cccDNA, but Knockdown of STAT3 resulted in the opposite effect, in which overexpression of DTX4 or MSL2 could rescue the levels of HBV cccDNA in HepG2-NTCP cells-infected HBV (Figure 7F and S7C). Notably, the ubiquitination experiment showed that STAT3 increased the ubiquitin modification of APOBEC3B by E3 ubiquitin ligase DTX4 or MSL2 in HepG2 cells (Figure 7G and S7D). Functionally, overexpression of DTX4 or MSL2 could rescue the levels of HBV cccDNA-mediated by siSTAT3 in HepG2-NTCP cells-infected HBV, while knock down of DTX4 or MSL2 could attenuate the levels HBV cccDNA-mediated by STAT3 overexpression in the cells (Figure 7H and S7E-G). Meanwhile, ChIP-qPCR assays indicated that the above treatments affect the binding of APOBEC3B to HBV cccDNA in the system (Figure 7I and S7H), suggesting that STAT3 confers HBV cccDNA stability by increasing ubiquitination degradation of APOBEC3B through DTX4 and MSL2 and decreasing the binding of APOBEC3B to HBV cccDNA. We conclude that STAT3 enhances the stability of HBV cccDNA and HBV replication through DTX4/MSL2-mediated ubiquitination of APOBEC3B.

### Cucurbitacin I limits the stability of HBV cccDNA and HBV replication by inhibiting p-STAT3/DTX4/MSL2/APOBEC3B signalling

**Figure 8:**
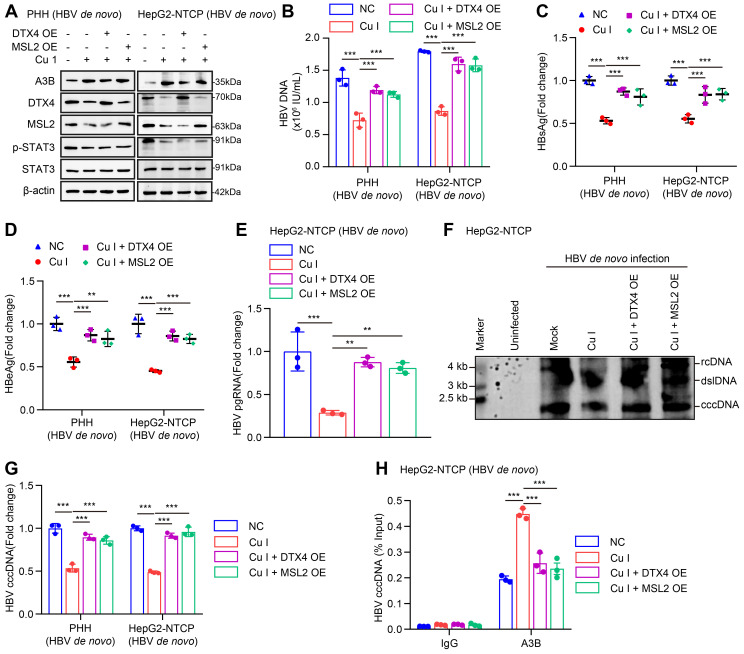
Cucurbitacin I limits the stability of HBV cccDNA and HBV replication by inhibiting p-STAT3/DTX4/MSL2/APOBEC3B signalling. (A-H) PHH and HepG2-NTCP cells were subjected to HBV infection (MOI = 500) and treated with factors as indicated at 1 and 4 dpi (days post-infection). Analysis was performed 7 days post-infection. The protein expression was verified by Western blot analysis in the cells (A). The HBV DNA content was tested by real-time PCR (B). The levels of HBsAg and HBeAg were measured by ELISA as appropriate in the supernatants (C, D). The levels of HBV pgRNA were determined by RT-qPCR in the cells (E). The HBV cccDNA content was examined by Southern blot analysis (F) and qPCR analysis (G). The binding levels of APOBEC3B to HBV cccDNA micro-chromosome were evaluated by ChIP-qPCR analysis in the cells (H). The mean ± SD of at least three experiments is shown. Statistically significant differences are indicated as follows: ^**^P < 0.01, ^***^P < 0.001. Abbreviation: Cu I, Cucurbitacin I, A3B, APOBEC3B.

Given that cucurbitacin I is an inhibitor of p-STAT3, we concern the significance of cucurbitacin I in anti-HBV therapy. According to our data, we supposed that cucurbitacin I might limit the stability of HBV cccDNA and HBV replication by inhibiting p-STAT3/DTX4/MSL2/APOBEC3B signalling. Furthermore, Western blot analysis showed that overexpression of DTX4 or MSL2 could block the cucurbitacin I-elevated APOBEC3B by targeting p-STAT3 in HBV-infected PHH and HepG2-NTCP cells (Figure 8A and S8A). Functionally, overexpression of DTX4 or MSL2 could rescue the attenuating effect of cucurbitacin I on the levels of HBV DNA, HBsAg, HBeAg in HBV-infected PHH and HepG2-NTCP cells (Figure 8B-E), and on the levels of HBV pgRNA and HBV cccDNA in HBV-infected PHH and HepG2-NTCP cells (Figure 8F-G). ChIP-qPCR assays indicated that the overexpression of DTX4 or MSL2 blocked the increasing effect of cucurbitacin I on levels of APOBEC3B binding to the HBV cccDNA (Figure 8H). Thus, we conclude that cucurbitacin I limits the stability of HBV cccDNA and HBV replication by inhibiting p-STAT3/DTX4/MSL2/APOBEC3B signalling.

## Discussion

The persistence of HBV cccDNA in hepatocytes is the major obstacle in anti-HBV therapies 10-12. At present, it is urgent to identify the targets of HBV cccDNA for eradication of HBV in liver. Based on that HBV activates STAT3 signalling in liver cells to support virus replication 52, 53, we supposed that STAT3 might be served as a target. However, underlying mechanism of HBV cccDNA modulation is unclear. In this study, we try to identify the novel therapeutical target of HBV cccDNA for improving the efficiency of anti-HBV.

Given that cucurbitacin I is an inhibitor of p-STAT3 in cancer therapy, we performed RNA-Seq analysis using HBV-infected HepG2-NTCP cells treated with cucurbitacin I. Interestingly, RNA-Seq analysis showed that cucurbitacin I displayed a wide range of biological functions in liver cancer, in which cucurbitacin I was associated with viral diseases. Then, we verified that the STAT3-targeting inhibitor cucurbitacin I could eliminate the levels of HBV cccDNA and HBV replication in cells. Particularly, cucurbitacin I could enhance the sensibility of PEG-IFN α against HBV *in vitro* and* in vivo*.

It has been reported that APOBEC3A and APOBEC3B contribute to the specific degradation mechanism of HBV cccDNA by interaction with nuclear cccDNA, resulting in the cytidine deamination, apurinic/apyrimidinic site formation, and finally cccDNA degradation that prevented HBV reactivation 26. To better understand the mechanism by which cucurbitacin I limited the levels of HBV cccDNA, we concerned whether cucurbitacin I affected APOBEC3A and APOBEC3B in cells. Strikingly, we observed that cucurbitacin I enhanced the binding of APOBEC3B to HBV cccDNA by blocking the degradation of APOBEC3B, but not APOBEC3A. It is implied that cucurbitacin I may reduce the proteasome degradation of APOBEC3B by E3 ubiquitin ligase. Then, we identified that cucurbitacin I significantly down-regulated the expression of E3 ubiquitin ligase DTX4 and HECW1 in cells according to the RNA-Seq analysis. Then, we validated that DTX4, but not HECW1, was able to modulate HBV cccDNA and HBV replication in our system. Mechanistically, we found that DTX4 reduced the binding of APOBEC3B to HBV cccDNA by increasing the K48 ubiquitination degradation of APOBEC3B, leading to the stability of HBV cccDNA. It suggests that cucurbitacin I limits the stability of HBV cccDNA by modulating E3 ubiquitin ligase DTX4 to block the degradation of APOBEC3B in liver. Next, we identified that p-STAT3 transcriptionally contributed to the activation of DTX4 by binding to the 5' flanking region (-949 to -939 bp) of *DTX4* gene promoter region in cells.

Our previous report showed that HBx-elevated MSL2 modulated HBV cccDNA by inducing degradation of APOBEC3B 23. Thus, we were interested in whether p-STAT3 transcriptionally activated MSL2 as well to synergistically regulate HBV cccDNA with DTX4. Our data demonstrated that DTX4/MSL2 independently and synergistically degraded APOBEC3B by ubiquitination to increase the stability of HBV cccDNA. Further, we validated that p-STAT3 promotes the transcription of MSL2 by binding to the 5' flanking region (-852 to -842 bp) of *MSL2* gene promoter region. It suggests that p-STAT3 transcriptionally activates both DTX4 and MSL2 to enhance the levels of HBV cccDNA in cells, synergistically. Therefore, our finding implies that any inhibitors targeting p-STAT3 are available to control HBV cccDNA. Clinically, we showed that the levels of HBV were positively correlated with those of p-STAT3 in human liver samples. It has been reported that the small protein of HBsAg expression in HCC cells induces endoplasmic reticulum (ER) stress that stimulates the activating transcription factor 4 (ATF4) to increase the expression and secretion of fibroblast growth factor 19 (FGF19), which in turn activated JAK2/STAT3 signalling 53. We validated that HBV-elevated p-STAT3 contributed to the stability of HBV cccDNA and HBV replication by promoting the ubiquitination degradation of APOBEC3B mediated through both DTX4 and MSL2 in liver in a positive feedback manner, keeping high levels of HBV. Thereby, p-STAT3 is a crucial target to cut off the loop.

Considering that MSL2 is not reflected in RNA-Seq data and there is limitation of RNA-Seq, we further tested the effect of cucurbitacin I on MSL2. Mechanically, we validate that cucurbitacin I could reduce the expression of DTX4 and MSL2, and enhance the protein levels of APOBEC3B, which increased the levels of APOBEC3B binding on the HBV cccDNA in cells, leading to reduce the stability of HBV cccDNA and HBV replication. Accumulating evidence suggests that IFN-α inhibits the levels of the HBV cccDNA 30-37, and it has been reported that IFN-α up-regulates APOBEC3A cytidine deaminases 26. Our finding showed that cucurbitacin I reduced the stability of HBV cccDNA through APOBEC3B. Therefore, the combination of cucurbitacin I and PEG-IFN α should result in the elimination of APOBEC3A and APOBEC3B binding to the HBV cccDNA, leading to more decrease of stability of HBV cccDNA. Surprisingly, we validated that cucurbitacin I enhanced the sensitivity of PEG-IFN α against HBV *in vitro* and* in vivo*. Thus, we conclude that STAT3-targeting inhibitor cucurbitacin I limits the stability of HBV cccDNA and HBV replication in liver by inhibiting p-STAT3/DTX4/MSL2/APOBEC3B signalling.

Taken together, we summarized a model that HBV-elevated p-STAT3 confers the stability of HBV cccDNA and HBV replication in a positive feedback manner through DTX4/MSL2/APOBEC3B signalling in liver. The p-STAT3 is able to transcriptionally activate E3 ubiquitin ligase DTX4/MSL2 in cells, which enhances the ubiquitination degradation of APOBEC3B and decreases the binding of APOBEC3B to HBV cccDNA, leading to the increase of the stability of HBV cccDNA and HBV replication. It suggests that p-STAT3 is a crucial therapeutical target of HBV cccDNA. Consistently, p-STAT3 targeting inhibitor cucurbitacin I displays the role of limiting HBV cccDNA by inhibiting the p-STAT3/DTX4/MSL2/APOBEC3B signalling. Moreover, the cucurbitacin I enhances the sensitivity of PEG-IFN α against HBV by synergistically modulating APOBEC3B and APOBEC3A in liver.

## Supplementary Material

Supplementary materials and methods, figures and tables.

## Abbreviations

PEG-IFN: pegylated interferon
MSL2: Male-specific lethal 2
HBV: Hepatitis B virus
HCC: hepatocellular carcinoma
HBV cccDNA: HBV covalently closed circular DNA
APOBEC3A: apolipoprotein B mRNA editing enzyme, catalytic polypeptide-like 3A
APOBEC3B: apolipoprotein B mRNA editing enzyme, catalytic polypeptide-like 3B
HIF1α: hypoxia inducible factor-1 α
NF-κB: nuclear factor kappa-B
IFN-α: Interferon-α
ISGs: IFN-stimulated genes
DTX: Deltex
ALKBH5: AlkB homolog 5
TYK2: Tyrosine Kinase 2
HDI: Hydrodynamic injection
ANOVA: analysis of variance
CHX: cycloheximide
LIHC: liver hepatocellular carcinoma
ER: endoplasmic reticulum
ATF4: activating transcription factor 4
FGF19: fibroblast growth factor 19
