# Supplementary material for: p-STAT3-elevated E3 ubiquitin ligase DTX4 confers the stability of HBV cccDNA by ubiquitinating APOBEC3B in liver
Source: Theranostics. 2024 Sep 16;14(15):6036–52. doi: 10.7150/thno.99407 (PMC11426250; doi:10.7150/thno.99407)
Supplement: Supplementary file 1 — Supplementary materials and methods, figures and tables. [file thnov14p6036s1.pdf]

## Materials and methods

### Cell culture, HBV production, infection and transfection

Cell lines such as HEK 293T, HepG2, HepG2-NTCP and HepG2.2.15 were maintained in Dulbecco's modified Eagle's medium (DMEM) supplemented with 10% fetal bovine serum (FBS) [1]. HepG2-NTCP and HepG2.2.15 cells were cultured in 400 μg/mL G418. HepG2 cells were transfected with the hNTCP expression plasmid using TransIT-LT1 (Mirus, USA), and HepG2-NTCP cells were generated according to the manufacturer's instructions. HepAD38 (tet-off) cells were cultured in DMEM/F12 medium supplemented with 10% FBS and 400 μg/mL G418 (for induction of HBV replication). Primary human hepatocytes (PHHs) were purchased from Shanghai RILD Inc. (Shanghai, China). These cells were cultured in a similar manner using the same plating strategy and incubation medium as previously described [2, 3]. All cell lines were treated with 100 U/mL penicillin and 100 mg/mL streptomycin and incubated in a humidified incubator equilibrated with 5% $CO_2$ at 37 °C. Cells were cultured in a 100 mm dish, 6-well plate or 24-well plate for 12 h and then transfected with plasmids, siRNAs, or their corresponding negative controls (unless otherwise indicated, a concentration of 100 nM was used for siRNA transfection, and cells were transfected with 10, 2, or 0.5 μg of plasmids in a 100 mm dish, 6-well plate or 24-well plate). Transfection was performed using Lipofectamine 3000 reagent (Invitrogen, Carlsbad, CA, USA) according to the manufacturer's protocol.

### HBV particle collection and infection

The HBV particles used in this study were mainly derived from HepAD38 cells, and prepared and quantified by a previously described method [4, 5]. HBV infection was performed as previously described [2, 3, 5].

**RNA extraction and quantitative real-time PCR (RT–qPCR)**

Total RNA was extracted from cells (or liver tissues from human liver chimeric mice) using TRIzol reagent (Solarbio, Beijing, China). First-strand cDNA was synthesized using Hifair III 1st Strand cDNA Synthesis SuperMix (Yeasen Biotech, Shanghai, China). Quantitative real-time PCR was performed in a StepOnePlus real-time PCR system (Bio–Rad) using SYBR Green qPCR Master Mix (Yeasen Biotech, Shanghai, China). Relative fold differences in transcription were calculated by the $2^{-\Delta\Delta Ct}$ method. GAPDH was used as the internal control for normalization. The primers used are listed in Supporting Information Table 4.

**Dual-luciferase reporter assay**

Cells were transferred into 24-well plates at $3\times 10^4$ cells/well. After 12 h, the cells in each well were transiently co-transfected with 0.2 μg of the pRL-TK plasmid (Promega, Madison, WI, USA) containing the Renilla luciferase gene used for internal normalization and the pGL3-DTX4 promoter, pGL3-MSL2 promoter, pGL3-DTX4 promoter mut or pGL3-MSL2 promoter mut plasmid (lost the ability to interact with STAT3, termed mutant luciferase reporter) (Supporting Information Table 5). Luciferase activities were measured as previously described [6]. All experiments were performed at least three times.

**HBV cccDNA extraction and quantification**

The procedure for the extraction of HBV cccDNA from HepAD38 (tet-off) has been reported previously [4, 5]. Briefly, aliquots of each DNA sample extracted from cell pellets were treated for 1 hour at 37 °C with 10 U of Plasmid-Safe ATP-Dependent DNase (Epicentre, Madison, WI). qPCR analysis was carried out in a Mastercycler ep realplex instrument (Eppendorf, Germany) to quantify the cccDNA. The primers used in this experiment are listed in Supporting Information Table 4.

**Quantification of HBV antigens and serum HBV DNA**

Hepatitis B virus surface antigen (HBsAg) and hepatitis B virus e antigen (HBeAg) in culture supernatants were assayed with commercial enzyme-linked immunosorbent assay (ELISA) kits (Kehua Bioengineering, Shanghai, China). HBV DNA in the supernatants of HepAD38 cells and HBV-infected HepG2-NTCP cells was quantified by using a diagnostic kit with a lower limit of detection of 400 copies/mL (Sansure Biotech, Changsha, China).

**Immunofluorescence analysis**

Cells in 6-well plates were washed three times with precooled PBS, fixed with 4% paraformaldehyde for 10 min, and permeabilized with 0.5% Triton X-100 for 10 min at room temperature. After incubation for 1 h with 5% BSA to block nonspecific binding, primary

antibodies were added for incubation overnight at 4 °C. The bound antibodies were detected by incubation with secondary antibodies. Images were acquired using fluorescence microscopy. The antibodies used for immunofluorescence analysis are listed in Supporting Information Table 6.

**Co-immunoprecipitation (Co-IP)**

Whole-cell extracts were prepared using IP lysis buffer (Beyotime) supplemented with a complete protease inhibitor cocktail (Solarbio) according to the manufacturer's instructions. Cell lysates were then incubated with the IP antibody overnight at 4 ℃ on a rotating wheel before they were incubated with Protein A/G magnetic beads (Millipore) for 4 h at 4 ℃ on a rotating wheel. The PPIs were analysed by immunoblotting.

**Immunohistochemical (IHC) staining**

Mice were sacrificed, and their liver tissues were fixed and embedded in paraffin. First, the sections were dewaxed. Then, antigen retrieval was performed at 95 °C with citrate buffer (pH 6.0) for 15 min. The slides were treated with 3% $H_2O_2$ for 10 min and blocked with goat serum for 1 h. Then, the slides were incubated with a monoclonal antibody at 4 ℃ overnight. After washing three times with 0.01 M PBS, the sections were incubated with horseradish peroxidase-conjugated goat anti-rabbit or anti-mouse IgG (OriGene, Beijing, China) for 30 min at 37 °C. Immunohistochemistry staining was performed using the chromogen 3,3′-diaminobenzidine (DAB), and counterstaining was performed with Mayer's

haematoxylin (ZSGB-BIO, China). The sections were then dehydrated and covered with a coverslip. The antibodies used for IHC staining are listed in Supporting Information Table 6.

**Chromatin immunoprecipitation (ChIP)**

ChIP was performed by using a SimpleChIP® Plus Sonication CHIP Kit 4C and RT reagents according to the manufacturer's instructions (Cell Signaling Technology, MA, USA) [7]. DNA synthesis was analyzed by quantitative PCR. Precipitated DNA was analyzed by quantitative PCR, and the primers used are listed in Supporting Information Table 4.

**TCGA database**

Public TCGA (https://portal.gdc.cancer.gov/) data repositories for liver hepatocellular carcinoma (LIHC) (Cancer Genome Atlas Network, 2014) were used for analysis of STAT3 and MSL2 expression.

**Western blot analysis**

Total protein was extracted by lysis of hepatoma cells or tissues with RIPA buffer. Protein concentrations were measured using the Bradford assay, and 20-50 μg of protein extracts were subjected to SDS–PAGE. Then, proteins were transferred to a nitrocellulose membrane, blocked with 5% nonfat milk and incubated with primary antibodies overnight at 4 °C. After incubation with an anti-mouse (1:10,000) or anti-rabbit (1:10,000) secondary antibody for 1 h

at 37°C, immunocomplexes were visualized with Super ECL Detection Reagent (Yeasen Biotech, Shanghai, China). The primary antibodies used for Western blot analysis are listed in Supporting Information Table 6.

**RNA-Seq analysis**

The cells were collected in TRIzol reagent, and RNA-Seq analysis was performed at Shanghai Majorbio Bio-pharm Technology Co. Ltd. The data were analyzed on the free online platform Majorbio Cloud Platform (https://www.majorbio.com/). Genes with an FDR-adjusted P < 0.05 were considered significantly differentially expressed.

**Bioinformatics analysis**

The public web servers JASPAR (https://jaspar.elixir.no/) was used to conduct bioinformatics analysis, JASPAR was applied to the binding sites of transcription factor on the STAT3, DTX4 or MSL2 promoter.

## References


1. Wang ZY, Li YQ, Guo ZW, Zhou XH, Lu MD, Xue TC, et al. ERK1/2-HNF4α axis is involved in epigallocatechin-3-gallate inhibition of HBV replication. Acta Pharmacol Sin. 2020; 41: 278-85.
2. Ni Y, Urban S. Hepatitis B Virus Infection of HepaRG Cells, HepaRG-hNTCP Cells, and Primary Human Hepatocytes. Methods Mol Biol. 2017; 1540: 15-25.
3. Yang G, Feng J, Liu Y, Zhao M, Yuan Y, Yuan H, et al. HAT1 signaling confers to assembly and epigenetic regulation of HBV cccDNA minichromosome. Theranostics. 2019; 9: 7345-58.
4. Gao Y, Feng J, Yang G, Zhang S, Liu Y, Bu Y, et al. Hepatitis B virus X protein-elevated MSL2 modulates hepatitis B virus covalently closed circular DNA by inducing degradation of

APOBEC3B to enhance hepatocarcinogenesis. Hepatology. 2017; 66: 1413-29.

5. Zhao L, Yuan H, Wang Y, Geng Y, Yun H, Zheng W, et al. HBV confers innate immune evasion through triggering HAT1/acetylation of H4K5/H4K12/miR-181a-5p or KPNA2/cGAS-STING/IFN-I signaling. J Med Virol. 2023; 95: e28966.
6. Zhang T, Zhang J, You X, Liu Q, Du Y, Gao Y, et al. Hepatitis B virus X protein modulates oncogene Yes-associated protein by CREB to promote growth of hepatoma cells. Hepatology. 2012; 56: 2051-9.
7. Yang Y, Zhao X, Wang Z, Shu W, Li L, Li Y, et al. Nuclear Sensor Interferon-Inducible Protein 16 Inhibits the Function of Hepatitis B Virus Covalently Closed Circular DNA by Integrating Innate Immune Activation and Epigenetic Suppression. Hepatology. 2020; 71: 1154-69.

## Supplementary figure legends

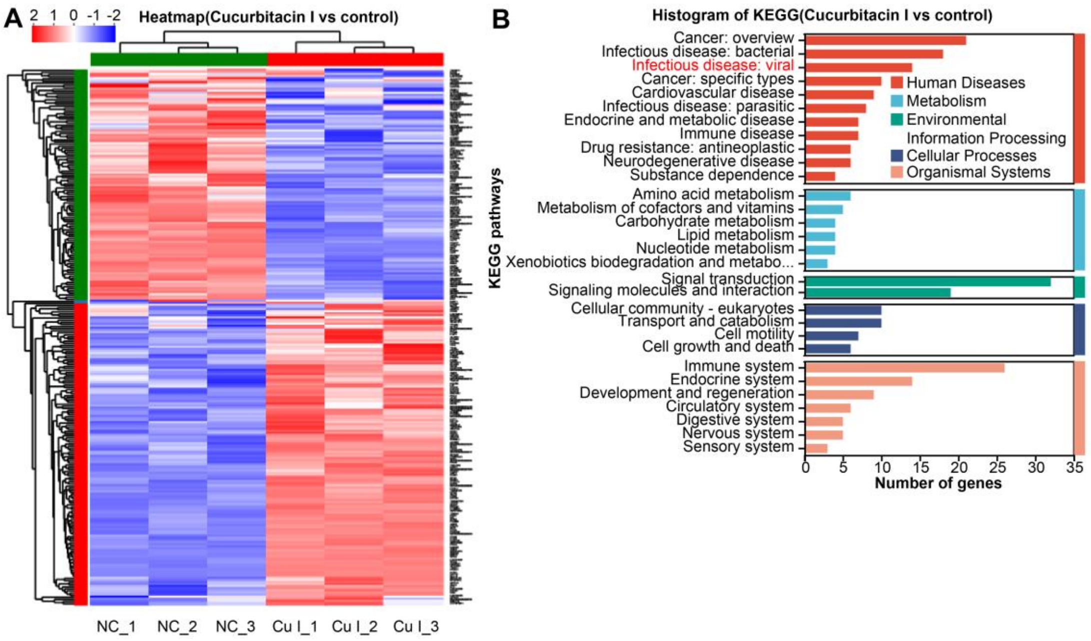


**FIGURE S1** RNA-Seq profiling of the effect of Cucurbitacin I on HBV-infected HepG2-NTCP cells. (A) Heatmap of RNA-Seq data from HBV-infected HepG2-NTCP cells treated with cucurbitacin I. (B) KEGG pathway annotation of the RNA-Seq data. The mean ± SD of at least three experiments is shown. Abbreviation: Cu I, Cucurbitacin I.

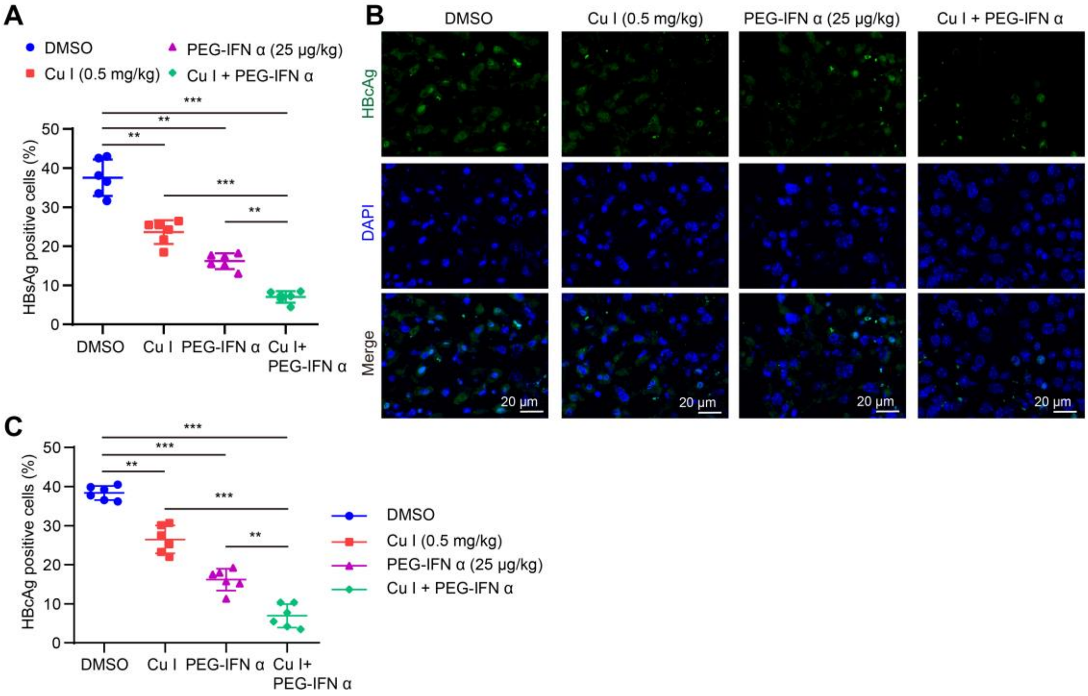

**FIGURE S2** Cucurbitacin I attenuates the HBV replication and enhances the sensitivity of PEG-IFN α against HBV *in vivo*. (A) The positive staining rate of HBsAg was assessed by immunohistochemical staining. The HBsAg expression levels in liver tissue samples were quantified by using the software of ImageJ. (B) The HBcAg expression levels were examined by fluorescent staining in liver tissues. Scale bars: 20 μm. (C) ImageJ was used to quantitate the protein expression levels in the fluorescent staining analysis. The mean ± SD of at least three experiments is shown. Statistically significant differences are indicated as follows: $^{**}P < 0.01$, $^{***}P < 0.001$. Abbreviation: Cu I, Cucurbitacin I.

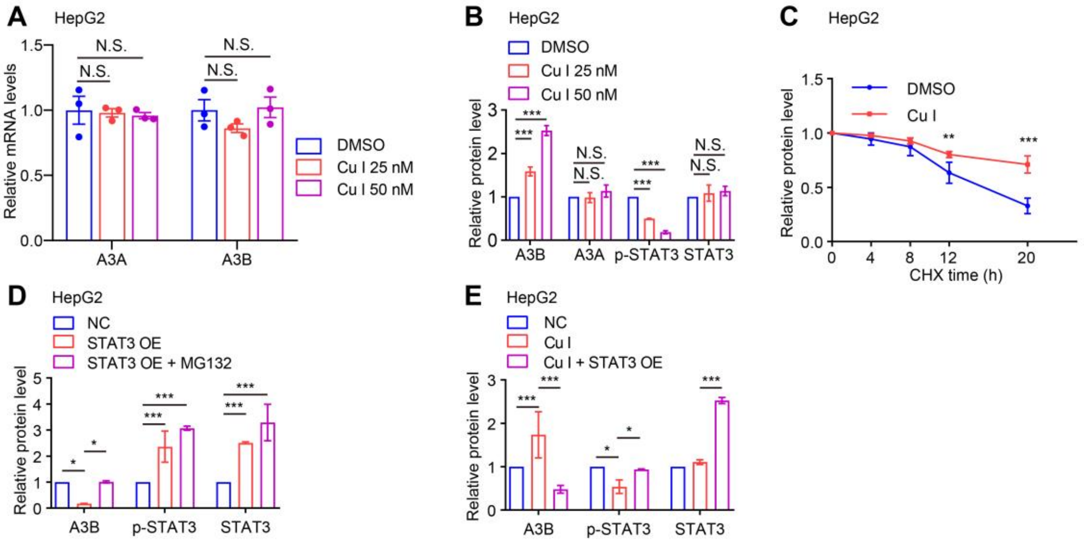


**FIGURE S3** The effect of Cucurbitacin I on APOBEC3A and APOBEC3B. (A) The mRNA expression levels of APOBEC3A and APOBEC3B were detected by RT-qPCR in HepG2 cells treated with cucurbitacin I. Cells treated with IL-6 to induce STAT3 activation for 16 hours. (B-E) ImageJ was used to quantitate the protein expression levels from Western blot analysis of Figure 3C, 3E-G. The mean ± SD of at least three experiments is shown. Statistically significant differences are indicated as follows: $^{*}P < 0.05$, $^{**}P < 0.01$, $^{***}P < 0.001$.

Abbreviation: N.S., not significant, Cu I, Cucurbitacin I, A3A, APOBEC3A, A3B, APOBEC3B.

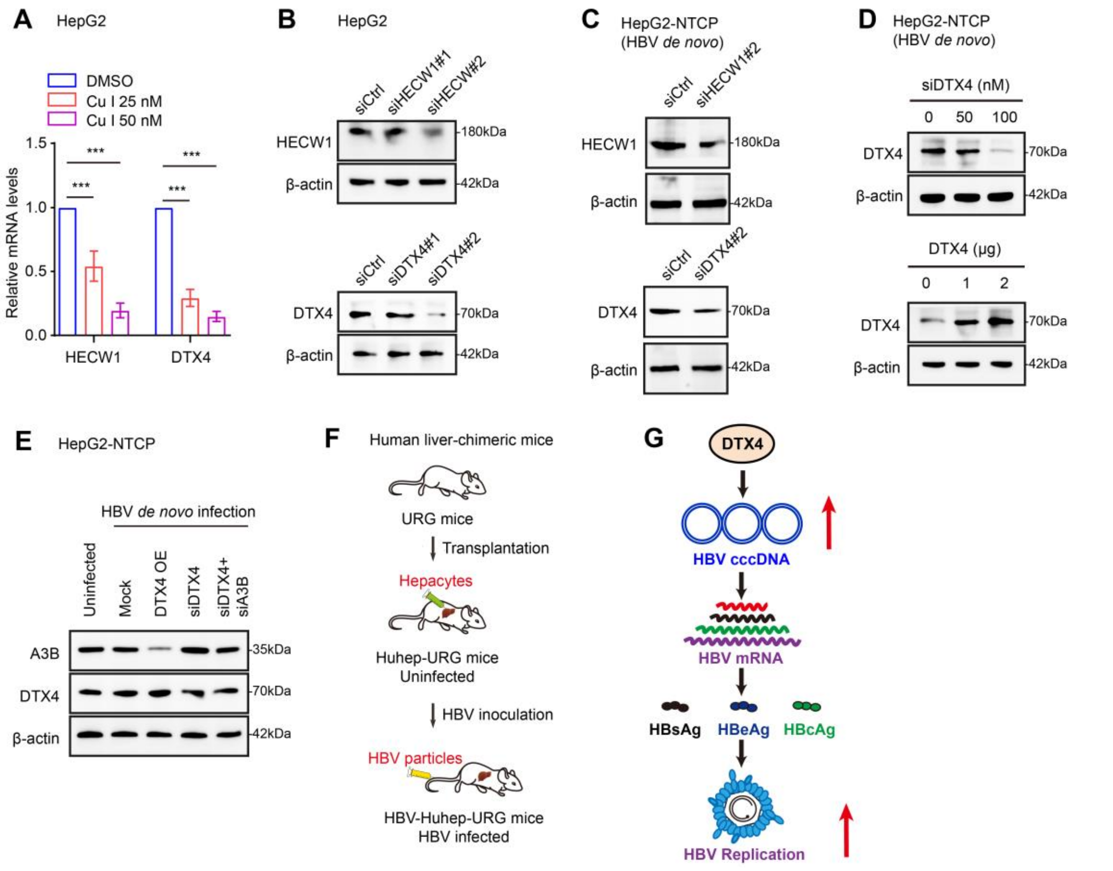


**FIGURE S4** E3 ubiquitin ligase DTX4 contributes to HBV cccDNA and HBV replication. (A) ImageJ was used to quantitate the protein expression levels from Western blot analysis of Figure 4B. (B, C) The interference efficiency of siDTX4 and siHECW1 were validated by Western blot analysis in HepG2 cells treated with IL-6 to induce STAT3 activation for 16 hours and HBV-infected HepG2-NTCP cells. (D) The interference and overexpression efficiency of DTX4 were verified by Western blot analysis in HBV-infected HepG2-NTCP cells. (E) The interference and overexpression efficiency of DTX4 and the interference efficiency of APOBEC3B were examined by Western blot analysis in HepG2-NTCP cells. (F) Schematic diagram of human liver-chimeric mouse model (Huhep-URG mouse model)

establishment. (G) A model of E3 ubiquitin ligase DTX4 inhibiting HBV cccDNA and HBV replication. The mean ± SD of at least three experiments is shown. Statistically significant differences are indicated as follows: $^{***}P < 0.001$. Abbreviation: A3B, APOBEC3B.

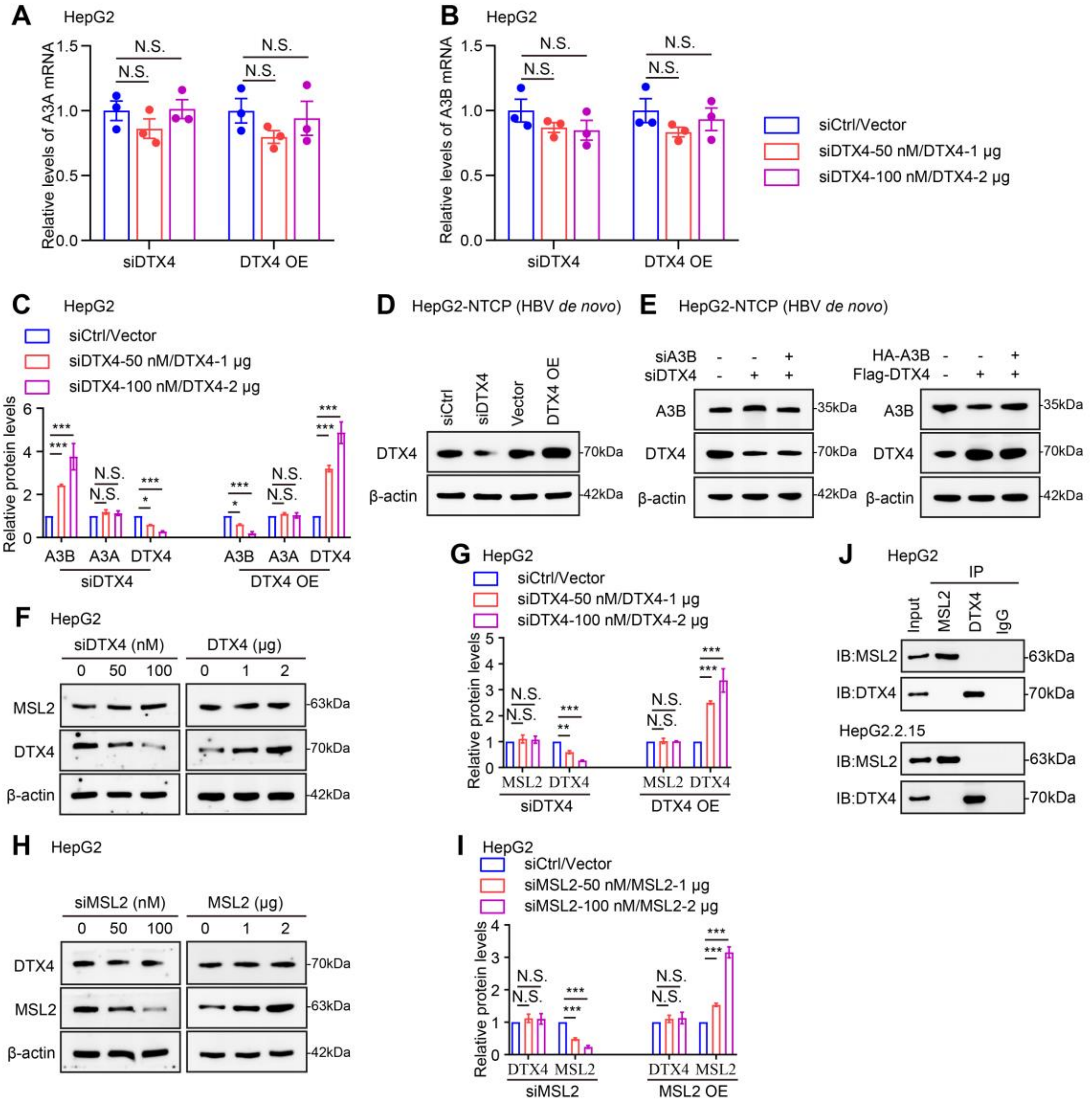


**FIGURE S5** The effect of DTX4 on APOBEC3A and APOBEC3B and the relationship between DTX4 and MSL2. (A, B) The mRNA expression levels of APOBEC3A or APOBEC3B were tested by RT-qPCR in HepG2 cells treated with knockdown or overexpression of DTX4. (C) ImageJ was used to quantitate the protein expression levels from Western blot analysis of Figure 5A. (D) The interference and overexpression efficiency

of DTX4 were evaluated by Western blot analysis in HBV-infected HepG2-NTCP cells. (E) The interference and overexpression efficiency of DTX4 and APOBEC3B were measured by Western blot analysis in HBV-infected HepG2-NTCP cells. (F) The effects of DTX4 on the expression levels of MSL2 were detected by Western blot in HepG2 cells. (G) ImageJ was used to quantitate the protein expression levels from Western blot analysis of Figure S5F. (H) The effects of MSL2 on the expression levels of DTX4 were detected by Western blot in HepG2 cells. (I) ImageJ was used to quantitate the protein expression levels from Western blot analysis of Figure S5H. (J) The binding affinity between endogenous DTX4 and MSL2 was evaluated by a Co-IP assay in HepG2 and HepG2.2.15 cells. The mean ± SD of at least three experiments is shown. Statistically significant differences are indicated as follows: $^{*}P < 0.05$, $^{**}P < 0.01$, $^{***}P < 0.001$. Abbreviation: N.S., not significant, A3B, APOBEC3B.

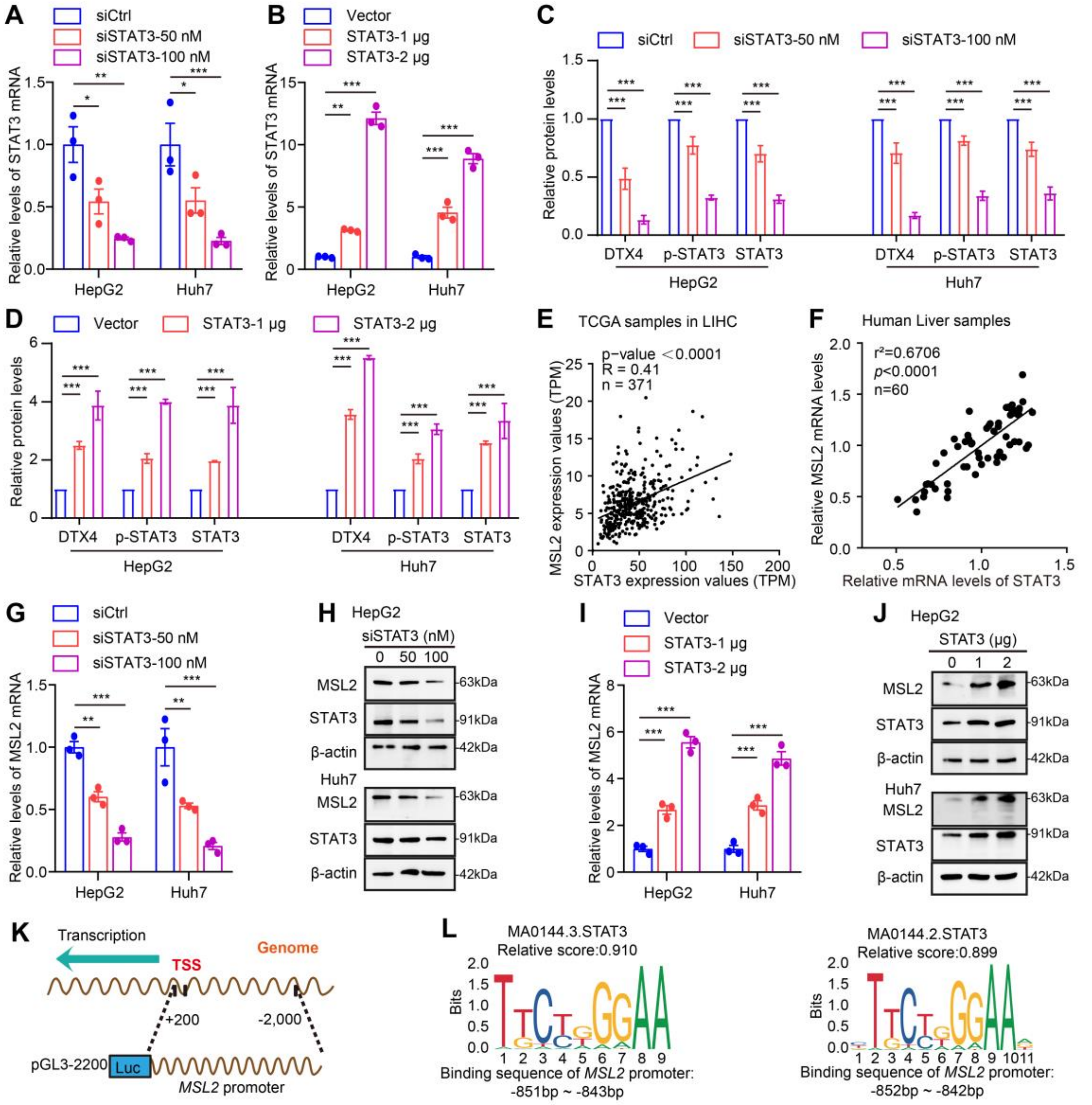


**FIGURE S6-1** STAT3 transcriptionally activates E3 ubiquitin ligase DTX4. (A, B) The interference and overexpression efficiency of STAT3 were detected by RT-qPCR in HepG2 and Huh7 cells treated with IL-6 to induce STAT3 activation for 16 hours. (C, D) ImageJ was used to quantitate the protein expression levels from Western blot analysis of Figure 6C and E. (E, F) Correlation analysis of the mRNA expression levels of STAT3 and MSL2 in the TCGA-LIHC (liver hepatocellular carcinoma) dataset (*n*=371) and paracancerous tissue of human clinical hepatocellular carcinoma samples (*n*=60). (G, H) The effects of STAT3 interference on MSL2 mRNA and protein expression levels were validated by RT-qPCR and

Western blot in HepG2 and Huh7 cells treated with IL-6 to induce STAT3 activation for 16 hours. (I, J) The effects of overexpression of STAT3 on MSL2 mRNA and protein expression were demonstrated by RT-qPCR and Western blot analysis in HepG2 and Huh7 cells treated with IL-6 to induce STAT3 activation for 16 hours. (K) Schematic illustration shows the pGL3-MSL2 promoter plasmid containing the -2,000 to +200 bp sequence of the 5' flanking region of MSL2. (L) STAT3 sequence motif logos and the binding elements in the promoter regions of MSL2 gene were identified by JASPAR database with a relative score higher than 80%. The mean ± SD of at least three experiments is shown. Statistically significant differences are indicated as follows: $^{*}P < 0.05$, $^{**}P < 0.01$, $^{***}P < 0.001$.

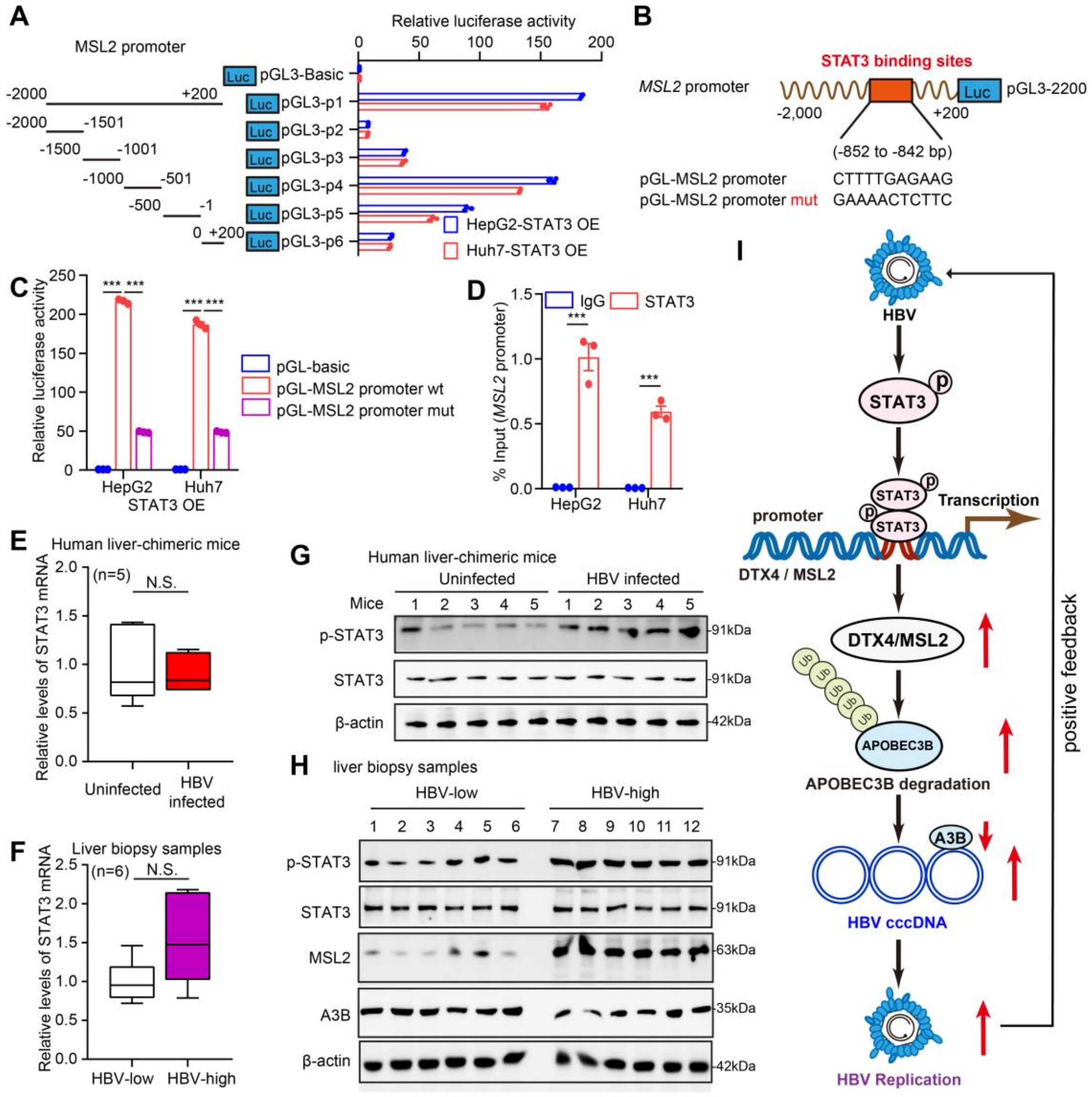


**FIGURE S6-2** STAT3 transcriptionally activates E3 ubiquitin ligase DTX4 and MSL2. (A) Luciferase activities of MSL2 promoter reporter gene vectors in HepG2 and Huh7 cells transfected with STAT3 were examined by dual-luciferase reporter assays. Cells treated with IL-6 to induce STAT3 activation for 16 hours. (B) A model demonstrates the predicted STAT3 binding site at -852 to -842 bp of the MSL2 promoter region. The generated mutant sites at the STAT3 binding site region were indicated. The wild-type MSL2 promoter (or mutant) was inserted into the upstream of the luciferase reporter gene in the pGL3-control vector. (C) Luciferase activities of MSL2 promoter wt or MSL2 mutant promoter reporter gene vectors in HepG2 and Huh7 cells transfected with STAT3 were examined by

dual-luciferase reporter assays. Cells treated with IL-6 to induce STAT3 activation for 16 hours. (D) The enrichment of STAT3 on the MSL2 region of -950 bp to -750 bp was examined by ChIP- qPCR assays in HepG2 and Huh7 cells treated with IL-6 to induce STAT3 activation for 16 hours. (E, F) The mRNA levels of STAT3 were analyzed by RT-qPCR analysis in liver tissues of HBV-infected (*n*=5) and uninfected (*n*=5) human liver chimeric mice and in liver biopsy specimens from clinical patients with a high HBV load (*n*=6) and with a low HBV load (*n*=6). (G) The protein levels of STAT3 and its phosphorylated levels were analyzed by Western blot analysis in liver tissues of HBV-infected (*n*=5) and uninfected (*n*=5) human liver chimeric mice. (H) The protein levels of STAT3, MSL2 and APOBEC3B, and the levels of p-STAT3 were evaluated by Western blot analysis in liver biopsy specimens from clinical patients with a high HBV load (*n*=6) and with a low HBV load (*n*=6). (I) A model of the mechanism by which HBV-elevated the transcription factor STAT3 promotes the transcription and expression of E3 ubiquitin ligases DTX4 and MSL2, thereby increasing the stability of HBV cccDNA and promoting HBV replication in a positive feedback manner. The mean ± SD of at least three experiments is shown. Statistically significant differences are indicated as follows: $^{***}P < 0.001$. Abbreviation: N.S., not significant, A3B, APOBEC3B.

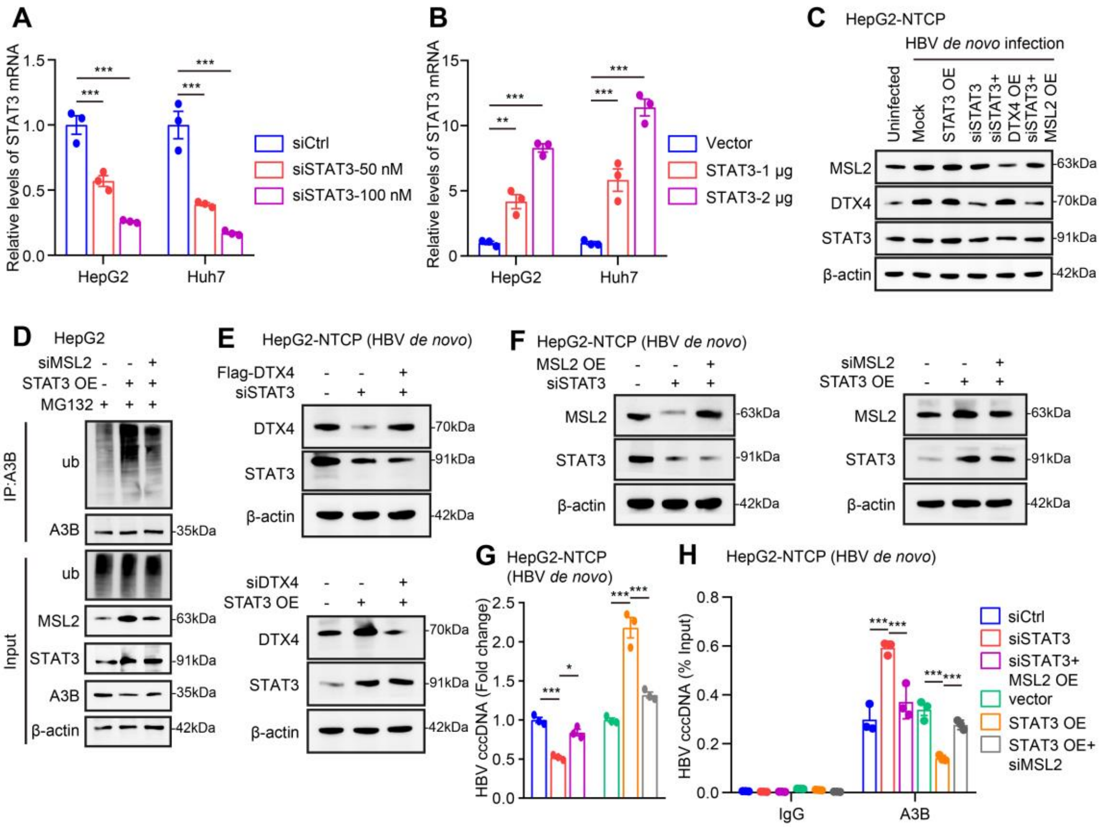


**FIGURE S7** STAT3 enhances the stability of HBV cccDNA and HBV replication through DTX4/MSL2-mediated ubiquitination of APOBEC3B. (A, B) The interference and overexpression efficiency of STAT3 were measured by RT-qPCR in HepG2 and Huh7 cells treated with IL-6 to induce STAT3 activation for 16 hours. (C) The overexpression efficiency of STAT3, DTX4 and MSL2 and the interference efficiency of STAT3 were detected by Western blot analysis in HepG2-NTCP cells. (D) Ubiquitination analysis of APOBEC3B in HepG2 cells when STAT3 were co-transfected with siMSL2 or not; cells were treated with MG132 (2 mM) for 24 h. Co-IP and Western blot analysis was performed to determine whether the effect of STAT3 on APOBEC3B protein expression through MSL2 in cells treated with IL-6 to induce STAT3 activation for 16 hours. (E, F) Interference and overexpression efficiency of DTX4, MSL2 and STAT3 were analyzed by Western blot analysis in HBV-infected HepG2-NTCP cells. (G, H) HepG2-NTCP cells were subjected to

HBV infection (MOI = 500) and treated as indicated at 1 and 4 dpi (days post-infection). Analysis was performed 7 days post-infection. qPCR analysis was performed to determine whether the effect of STAT3 on the level of HBV cccDNA through MSL2 in the cells (G). ChIP-qPCR analysis was performed to determine whether the effect of STAT3 on binding of APOBEC3B to HBV cccDNA micro-chromosome through MSL2 in the cells (H). The mean ± SD of at least three experiments is shown. Statistically significant differences are indicated as follows: $^{*}P < 0.05$, $^{**}P < 0.01$, $^{***}P < 0.001$. Abbreviation: A3B, APOBEC3B.

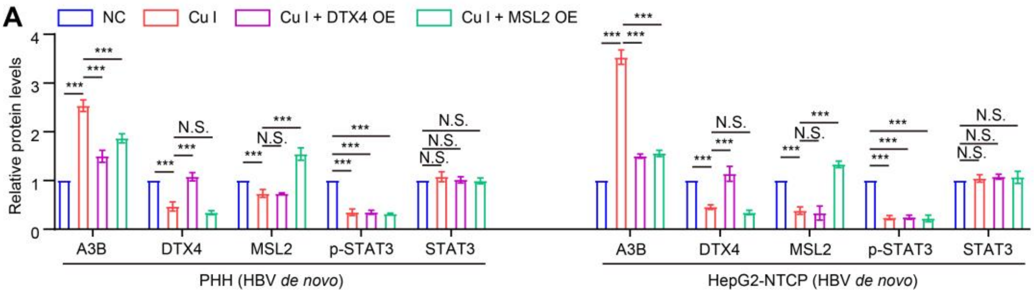


**FIGURE S8** Cucurbitacin I limits the expression of APOBEC3B by inhibiting p-STAT3/DTX4/MSL2 signalling. (A) ImageJ was used to quantitate the protein expression levels from Western blot analysis of Figure 8A. The mean ± SD of at least three experiments is shown. Statistically significant differences are indicated as follows: $^{***}P < 0.001$. Abbreviation: A3B, APOBEC3B, N.S., not significant.

## Supporting Information Tables

**Table 1.** The characteristics of patients with biopsy.

| Case No. | Age (year) | Gender | Diagnosis | Organ | HBV (copy/mL) |
|---|---|---|---|---|---|
| 1 | 55 | Male | NA | Liver | $< 5.6\times10^{2}$ |
| 2 | 70 | Male | NA | Liver | 0 |
| 3 | 67 | Male | NA | Liver | $< 5.6\times10^{2}$ |
| 4 | 74 | Male | NA | Liver | $< 5.6\times10^{2}$ |
| 5 | 68 | Male | NA | Liver | 0 |
| 6 | 65 | Male | NA | Liver | $< 5.6\times10^{2}$ |
| 7 | 58 | Male | Viral hepatitis type B | Liver | $8.29\times10^{7}$ |
| 8 | 58 | Male | Viral hepatitis type B | Liver | $1.57\times10^{8}$ |
| 9 | 68 | Male | Viral hepatitis type B | Liver | $1.93\times10^{7}$ |
| 10 | 36 | Male | Viral hepatitis type B | Liver | $1.08\times10^{7}$ |
| 11 | 59 | Male | Viral hepatitis type B | Liver | $6.89\times10^{5}$ |
| 12 | 57 | Male | Viral hepatitis type B | Liver | $2.60\times10^{5}$ |

Abbreviations: “NA” refers to not available.

**Table 2.** Clinical characteristics of 60 pairs of clinical liver cancer tissues.

| Clinicopathological features | | |
|---|---|---|
| | **No. of patients** | **Proportion (%)** |
| **Age** | | |
| ≤60 | 35 | 58.3 |
| >60 | 25 | 41.7 |
| **Gender** | | |
| Male | 50 | 83.3 |
| Female | 10 | 16.7 |
| **Cirrhosis status** | | |
| Yes | 32 | 53.3 |
| No | 28 | 46.7 |
| **Satellite nodule** | | |
| Positive | 14 | 23.3 |
| Negative | 46 | 76.7 |
| **Microvascular invasion** | | |
| M0 | 37 | 61.7 |
| M1 | 15 | 25 |
| M2 | 8 | 13.3 |
| **Differentiation degree** | | |
| Well-differentiated | 3 | 5 |
| Moderately-differentiated | 41 | 68.3 |
| Poorly-differentiated | 16 | 26.7 |

**Table 3.** The information of mice with humanized liver.

| Mice | HSA (μg/mL) | HBV DNA (×$10^6$ copy/mL) | HBsAg (IU/mL) | HBeAg (PEIU/mL) |
|---|---|---|---|---|
| Mice-Uninfected-1 | 1844 | 0 | 0 | 0 |
| Mice-Uninfected-2 | 1916 | 0 | 0 | 0 |
| Mice-Uninfected-3 | 2065 | 0 | 0 | 0 |
| Mice-Uninfected-4 | 3243 | 0 | 0 | 0 |
| Mice-Uninfected-5 | 4274 | 0 | 0 | 0 |
| Mice-HBV infected-1 | 1128 | 11.50 | 54.78 | 22.85 |
| Mice-HBV infected-2 | 3289 | 1.34 | 25.24 | 5.86 |
| Mice-HBV infected-3 | 3662 | 1.28 | 25.51 | 6.85 |
| Mice-HBV infected-4 | 1094 | 4.38 | 27.28 | 8.13 |
| Mice-HBV infected-5 | 1571 | 23.7 | 62.59 | 24.18 |

Abbreviations: “HSA” refers to “Human Serum Albumin”.

**Table 4.** List of Oligonucleotide Sequences used in this article.

| Gene | Primer | Sequence (5'-3') |
|---|---|---|
| **Primers for RT-qPCR** | | |
| GAPDH | Forward | GAGTCAACGGATTTGGTCGT |
| | Reverse | TTGATTTTGGAGGGATCTCG |
| DTX4 | Forward | TTAAGGCAGCCGTGGTCAATG |
| | Reverse | CTTCAGTGGGCCTCGAATGG |
| HECW1 | Forward | GTTTTGTGTCCTTGCCCACT |
| | Reverse | GAATTGCAGCTGTCCACTCA |
| MSL2 | Forward | ACAGTGAGAAAGTTCAGCCA |
| | Reverse | AGCACGCCCACATTTACA |
| APOBEC3A | Forward | GAGAAGGGACAAGCACATGG |
| | Reverse | TGGATCCATCAAGTGTCTGG |
| APOBEC3B | Forward | TGACCCTTTGGTCCTTCGAC |
| | Reverse | CATGGCGGCCGTAAAAGC |
| STAT3 | Forward | CAGCAGCTTGACACACGGTA |
| | Reverse | AAACACCAAAGTGGCATGTGA |
| mouse-gapdh | Forward | CCTGCCAAGTATGATGACAT |
| | Reverse | GTTGCTGTAGCCGTATTCA |
| mouse-dtx4 | Forward | TGTGCCTGTGAAAAACTTGAATG |
| | Reverse | TGGGATGGACTTTATCTCACTCT |
| mouse-stat3 | Forward | AGCTGGACACACGCTACCT |
| | Reverse | AGGAATCGGCTATATTGCTGGT |
| cccDNA | Forward | CGTCTGTGCCTTCTCATCTGC |
| | Reverse | GCACAGCTTGGAGGCTTGAA |
| HBV pgRNA | Forward | CTCCTCCAGCTTATAGACC |
| | Reverse | GTGAGTGGGCCTACAAA |
| GAPDH (ChIP) | Forward | TCGACAGTCAGCCGCATCT |
| | Reverse | CTAGCCTCCCGGGTTTCTCT |

| | | |
|---|---|---|
| cccDNA (ChIP) | Forward | CTCCCCGTCTGTGCCTTCT |
| | Reverse | GCCCCAAAGCCACCCAAG |
| DTX4-promoter (ChIP) | Forward | TAGCCCAATAAATTTTTAA |
| | Reverse | TTCAAAATTTAGTTGGGCT |
| MSL2-promoter (ChIP) | Forward | GGTTGGTCTCGCCCTCTTG |
| | Reverse | GTCCAACATTCAGAATCTA |
| **siRNA sequence** | | |
| DTX4#1 | | UCAAUUACGUAGCUAAAGCCA |
| DTX4#2 | | GGAUCGACCUCACUUCCAUUU |
| HECW1#1 | | UCAUCAAUGAGGUACAUGCCA |
| HECW1#2 | | GACCUCACUUUCACUGUUAAU |
| APOBEC3B | | CCUGAUGGAUCCAGACACA |
| STAT3 | | UCCAGUUUCUUAAUUUGUUGACGGGUU |
| MSL2 | | GUGCAUUAUAUCCCAGCAGAA |
| Control | | UUCUCCGAACGUGUCACGU |

**Table 5.** List of plasmids used in this article.

| Plasmids | Note |
|---|---|
| pcDNA3.1-vector | Vector |
| pcDNA3.1-HA-Vector | Vector |
| pCMV-Flag-Vector | Vector |
| pcDNA3.1-STAT3 | STAT3 (no tag) |
| pcDNA3.1-APOBEC3B | APOBEC3B (no tag) |
| pcDNA3.1-APOBEC3B-HA | HA-tagged APOBEC3B |
| pcDNA3.1-MSL2 | MSL2 (no tag) |
| pCMV-DTX4-Flag | Flag-tagged DTX4 |
| pCMV-ub WT-Flag | Flag-tagged ub WT |
| pCMV-ub K48R-Flag | Flag-tagged ub K48R |
| pCMV-ub K63R-Flag | Flag-tagged ub K63R |
| pGL3-Basic | Vector |
| pRL-TK | Vector |
| pGL3-P1 | DTX4 promoter -2000 ~ +200 |
| pGL3-P2 | DTX4 promoter -2000 ~ -1501 |
| pGL3-P3 | DTX4 promoter -1501 ~ -1001 |
| pGL3-P4 | DTX4 promoter -1001 ~ -501 |
| pGL3-P5 | DTX4 promoter -501 ~ -1 |
| pGL3-P6 | DTX4 promoter 0 ~ +200 |
| pGL3-DTX4 promoter mut | DTX4 promoter mutation |
| pGL3-P1 | MSL2 promoter -2000 ~ +200 |
| pGL3-P2 | MSL2 promoter -2000 ~ -1501 |
| pGL3-P3 | MSL2 promoter -1501 ~ -1001 |
| pGL3-P4 | MSL2 promoter -1001 ~ -501 |
| pGL3-P5 | MSL2 promoter -501 ~ -1 |
| pGL3-P6 | MSL2 promoter 0 ~ +200 |
| pGL3-MSL2 promoter mut | MSL2 promoter mutation |

**Table 6.** List of antibodies used in this article.

| Antibody | Manufacture | Catalog Number |
|---|---|---|
| HBc | Abcam | Ab115992 |
| APOBEC3A | Abclonal | A12399 |
| APOBEC3B | Abcam | ab184990 |
| p-STAT3 | Santa Cruz | sc-8059 |
| STAT3 | Santa Cruz | sc-8019 |
| HECW1 | proteintech | 24695-1-AP |
| DTX4 | proteintech | 25222-1-AP |
| Ubiquitin | Abcam | ab134953 |
| MSL2 | Novus | H00055167- D01P |
| HBsAg | Abcam | ab68518 |
| HBeAg | Abcam | ab8639 |
| Flag tag | MBL | M185-3L |
| Flag tag | CST | 14793S |
| β-actin | Sigma-Aldrich | A2228 |
